# A Hybrid Optical-Neural Network Approach for Micro-Vibration Image Shift Estimation in Space Cameras

**DOI:** 10.3390/s26216760

**Published:** 2026-10-26

**Authors:** Minlong Lian, Dawei Sang, Pu Wang, Xin Yi

**Affiliations:** School of Mechanics and Engineering Science, Peking University, Beijing 100871, China

**Keywords:** space camera, micro-vibration, image shift, lightweight neural network, model quantization, optical sensitivity

## Abstract

High-resolution space cameras require continuous knowledge of micro-vibration-induced image shift for image quality assessment and potential future compensation. This paper presents a *simulation-based feasibility study* of an optical measurement-assisted neural regression method that estimates imaging channel image shift without re-solving a full finite element model during inference. A high-frame-rate measurement channel shares the primary optics with the imaging channel; a lightweight, INT8-quantized and structurally pruned conventional neural regressor estimates the observable correction formed by the two measurement branch mirrors from rendered measurement frames, a calibrated sensitivity relation converts synchronized mirror-6 angular metrology into the same units, and both are added to the tracked measurement channel displacement. On the simulated dual-channel dataset, the proposed estimator reduces the correction RMSE from 0.396 px (centroid-only tracking) and 0.236 px (a specified structural-mismatch surrogate) to 0.130 px (0.133 px after INT8 quantization), which coincides with the structural scale of 0.132 px set by the common-path compensation residual; the remaining end-to-end image shift error of 0.398 px is dominated by the 0.378 px displacement tracking term rather than by the regressor. Its reported 0.0150 ms figure is a compute-only throughput estimate based on an assumed 120 GOPS INT8 accelerator; it excludes sensor exposure and readout, data transfer, synchronization, centroid tracking, and other pipeline overheads. A compute-only estimate does not establish the achievable end-to-end frame rate. The analytical error budget decomposes the measured shift error into displacement tracking, auxiliary metrology, and correction-scale terms and reproduces the measured value to within 1%. The modeled measurement branch does not observe independent motion of the imaging-only mirror; auxiliary state information or a validated mechanical constraint is required for a physically realizable estimator. The reported synthetic accuracy does not establish this observability; independent ray-tracing validation, optical bench experiments, hardware-in-the-loop tests, edge-hardware profiling, and on-orbit validation have not been performed.

## 1. Introduction

High-resolution space cameras form the core of modern Earth-observation systems, and their spatial resolution bounds the value of land-survey, disaster-monitoring, and mapping products [1,2]. On orbit, the payload is excited by micro-vibration from rotating machinery, including reaction wheels, cryocoolers, solar-array drives, and scanning mechanisms [3,4,5]. Reaction wheels are among the most prominent sources, and their disturbance spectra have been characterized through dedicated models and bench tests [6,7]. The disturbance energy, concentrated below a few hundred hertz [2,8], propagates through the spacecraft structure to the optical bench, where it induces rigid-body motion of the mirrors and, in turn, line-of-sight (LOS) jitter [9,10].

### 1.1. Background and Significance

During the detector integration time, LOS jitter appears as focal-plane image shift; the accumulated motion broadens the point-spread function and lowers the modulation transfer function (MTF) of the imaging chain [11,12,13,14]. For push-broom sensors that employ time-delay integration (TDI), a sub-pixel along-track shift already breaks the synchronization between scene motion and charge transfer and introduces geometric distortion [15,16]. Micro-vibration therefore degrades both the radiometric sharpness and the geometric accuracy of the imagery [10,14], which makes on-orbit characterization of the image shift important for quality assessment and for active compensation [1,17,18].

The prevailing estimation approach combines finite element analysis (FEA) with accelerometer measurements: a structural model propagates the measured disturbance to each mirror, and the resulting rigid-body motion is converted into image-plane shift through optical sensitivity matrices [19,20,21]. Integrated opto-mechanical modeling of this kind is mature for design-stage verification [9,19], yet it has two practical drawbacks. A full spacecraft FEA model is expensive to construct and must be revised whenever the structural configuration changes, and the model-to-reality discrepancy is non-negligible: full-chain analyses report LOS errors of 6–11% between simulation and ground or in-orbit tests [9,10]. The acceleration-to-shift mapping is therefore indirect, and its accuracy tends to decline as the structural dynamics evolve in orbit [5,17]. Isolation hardware reduces the transmitted disturbance [22,23], but it attenuates rather than measures the residual image shift and still leaves the instantaneous shift value unavailable to the imaging chain.

This paper develops an alternative that estimates the imaging channel image shift continuously from a dedicated optical measurement channel. The measurement channel shares the primary, secondary, tertiary, and quaternary mirrors with the imaging channel and then diverges to a high-frame-rate visible CMOS so that it observes the shared optics and its own branch while leaving normal imaging uninterrupted. Independent motion in the imaging-only branch is not directly observed. A lightweight, quantized neural network regresses the angular variations of the three non-shared mirrors directly from the measurement channel images, and the optical sensitivity relation (Section 2) converts these angles into the imaging channel shift. The proposed inference scheme avoids a full structural re-solve and is intended to support image quality assessment and possible future compensation without interrupting imaging. The present study uses synthetic opto-mechanical data (Section 3.3); it does not report an independently validated ray-tracing model or an on-board implementation. Carrying the trained regressor and calibrated sensitivities aboard is a proposed implementation route, subject to optical and hardware validation.

### 1.2. Related Works

The relevant literature can be grouped into four strands: opto-mechanical vibration analysis, image-based vibration measurement, on-board lightweight machine learning, and dual-channel shared-aperture optics.

#### 1.2.1. Opto-Mechanical Vibration Analysis

Integrated opto-mechanical modeling couples a structural finite element (FE) model with ray tracing so that the structural response is translated into line-of-sight (LOS) error or modulation transfer function (MTF) change. This paradigm underpinned the jitter analysis of the James Webb Space Telescope [19,24], whose wavefront sensing and control loop was validated against an integrated structural–optical model, and the jitter performance budgets of large space observatories [9,25]. At the sub-system level, sensitivity matrices derived from optomechanical constraint equations allow the dominant optical elements to be identified efficiently [21], full-chain studies on reaction wheel-excited systems validated the predicted LOS error against experiments within 6–11% [10], and jitter MTF under random loads was treated analytically [20]. More recently, the same integrated pipeline has been applied to the micro-vibration qualification of entire payloads, where the disturbance is traced from the reaction wheel through the supporting structure to the focal plane [1,14].

These methods are accurate and physically interpretable, but three limitations persist for on-orbit, real-time shift estimation. First, a full spacecraft FE model is expensive to construct and must be revised whenever the structural configuration changes [9,10]. Second, the model-to-reality discrepancy is non-negligible: full-chain analyses report LOS errors of 6–11% between simulation and ground or in-orbit tests [10], and this gap tends to grow as the structure ages and its dynamic modes drift. Third, and most important for the present problem, the FE-to-shift mapping is indirect and is intended for offline, design-time analysis; it does not provide an instantaneous estimate of the optical element state during operation, so the imaging chain cannot be compensated frame by frame from the model alone [5,17]. Isolation hardware partially addresses the source rather than the symptom: it attenuates the transmitted disturbance [22,23] but still leaves the instantaneous residual shift unavailable to the imaging channel.

#### 1.2.2. Image-Based Vibration and Blur Measurement

A second strand estimates vibration from the imagery itself, which is attractive because it needs no structural model. The relation between mechanical vibration and image blur has been characterized analytically through the vibration MTF and optical transfer function [11,12,13], and the resulting motion blur has been inverted by a long line of blind deconvolution methods: early sparse-prior formulations [26], normalized sparsity blind deconvolution [27], non-uniform shake deblurring [28], text-image $L_{0}$ deblurring [29], fast non-uniform camera shake removal [30], and the closely related evaluation framework of [31]; several of these were later unified by the fast motion deblurring formulation of [32]. In the space domain, jitter-degraded star images are restored by reconstructing the exposure trajectory [33], and dynamic point-spread-function models compensate for push-broom jitter under TDI [15,16]. Vision-based vibration measurement tracks a spot or a feature across frames and reaches sub-pixel accuracy [34,35], and learning-based centroid estimators improve the robustness of the measurement [36].

These methods share two limitations for the task addressed here. They measure the aggregate image motion on the focal plane, so they cannot separate the contributions of the individual non-shared mirrors—the information that the sensitivity relation of Section 2 requires in order to correct the imaging channel. And they act on the imaging channel after the exposure: deblurring is a post-processing step, and frame-by-frame tracking demands a high-frame-rate sensor, whereas the imaging channel of a space camera is typically exposed at a rate far below the disturbance bandwidth (10–200 Hz). A method that observes the disturbance on a *dedicated* high-rate channel, before it reaches the imaging focal plane, avoids both problems; this is precisely the role of the measurement channel proposed in this work.

#### 1.2.3. On-Board Lightweight Machine Learning

Deploying neural networks on board has become practical for resource-constrained platforms. CloudScout, a convolutional network for cloud screening on hyperspectral imagery, was flown on the PhiSat-1 CubeSat and demonstrated on-board inference under tight power and memory budgets [37]; the same family of on-board inference was later extended to event detection from multispectral data [38]. Compression techniques that combine pruning, trained quantization and Huffman coding [39], integer-only quantization [40], joint pruning and quantization [41], and efficient backbones such as MobileNetV2 [42] enable such deployment, and a general lightweight-design framework coupling knowledge distillation, pruning, and quantization was recently proposed for on-orbit image interpretation networks [43]. Taken together, these works establish that a network of order $10^{4}$–$10^{5}$ parameters can execute within the power and memory envelope of a spacecraft processing unit.

Despite this progress, on-board learning has been applied mainly to classification, detection, and segmentation, where the output is a label or a bounding box. Regressing the instantaneous angular state of optical elements from raw image data—the task considered here—has received little attention [37,43], and two additional constraints have not been addressed by existing on-board vision work. The measurement stream arrives at 2000Hz, so the per-frame budget is below 0.5ms; and the regression target is a continuous vector in microradian units whose error must stay below the sub-pixel shift it implies. The combination of a tight real-time budget and a high-precision continuous output is what distinguishes the present network from the classification focus of prior on-board deployments.

#### 1.2.4. Dual-Channel and Shared-Aperture Optics

Shared-aperture designs reuse the front optics for two channels and are adopted to reduce volume and mass. Dual-channel common-aperture spectrometers split the shared telescope output into two spectral channels [44], and multi-DOF optical-path sensitivity analyses of four-mirror telescopes provide the framework for translating mirror motion into optical-path change [45]. These systems share the aperture to deliver more spectral or field information, not to observe a disturbance: the two branches are co-aligned and carry the same scene, so the vibration-induced shift of one branch cannot be decoupled from the other [44,45]. A co-boresighted dual-channel layout is therefore a distinct concept. After the shared front end, the beam must diverge deliberately so that one branch continues to imaging and the other is routed to a dedicated high-rate measurement sensor; only then does the difference between the two channels isolate the non-shared-mirror contribution, which is the configuration introduced in Section 2. To the best of our knowledge, no published shared-aperture system uses one channel as a vibration metrology branch for the other, which is the gap this paper addresses at the optical level.

In summary, each strand leaves part of the problem open. FEA-based integrated models are commonly evaluated off-line and may retain a model-to-reality gap; image-based methods act on the imaging signal and generally do not identify individual mirror states; and existing shared-aperture systems do not use a dedicated measurement branch to infer the motion of non-shared elements. This study therefore examines, in simulation, whether a dual-channel optical layout combined with a conventional lightweight neural regressor can reconstruct the imaging channel shift without an on-board structural re-solve. The proposed architecture and its assumptions are developed in Section 2 and Section 3, analyzed in Section 4, and evaluated on synthetic data in Section 5. No optical bench, hardware-in-the-loop, or on-orbit validation is claimed.

### 1.3. Contributions

The main contributions of this paper are summarized as follows.

- We propose a dual-channel optical layout for space cameras in which a measurement channel shares the primary, secondary, tertiary, and quaternary mirrors with the imaging channel and then diverges to a high-frame-rate visible CMOS, so that the vibration-induced image shift is observed without interrupting normal imaging (Section 2).
- We formulate the imaging channel shift as the measurement channel shift plus the angular variations of the three non-shared mirrors (infrared folding mirror, image shift switching mirror, and image shift folding mirror) scaled by their vibration sensitivities, yielding a recovery rule that does not require a full structural re-solve during inference (the regressor is trained off-line on simulated opto-mechanical data; Section 2).
- We examine a lightweight, quantized three-output neural regressor in a synthetic setting. Its image-only input does not resolve the physical observability limitation identified in Section 2.3; we use INT8 quantization and pruning to study compatibility with a nominal on-board compute budget (Section 3).
- We derive an end-to-end estimation error bound that combines centroid noise, network inference error, and sensitivity uncertainty, and we state the conditions under which the simulated estimator remains accurate (Section 4).
- We evaluate the method on a simulated dataset against FEA-only, centroid-only, and ablated variants, reporting angle and image shift accuracy, robustness to sensor noise, and model complexity (Section 5).

### 1.4. Paper Organization

The remainder of this paper is organized as follows. Section 2 introduces the system model and preliminaries, including the notation, the dual-channel optical layout, and the optical sensitivity relation that connects the measurement and imaging channels. Section 3 presents the proposed hybrid method, comprising the measurement pipeline, the lightweight quantized network, and the end-to-end estimation algorithm. Section 4 derives the estimation error bound and discusses the assumptions and limitations of the method. Section 5 reports the numerical experiments, including the dataset, baselines, metrics, accuracy, robustness, ablation, and sensitivity studies. Section 6 concludes the paper and outlines directions for future work.

## 2. System Model and Preliminaries

This section introduces the notation used throughout the paper, describes the dual-channel optical layout of the space camera, and derives the optical sensitivity relation that connects the measurement channel to the imaging channel.

### 2.1. Notation

Table 1 summarizes the main symbols. The sets *C*, *M*, and *I* denote the shared (common) optical elements, the elements exclusive to the measurement channel, and the elements exclusive to the imaging channel, respectively. All angular quantities are measured relative to the nominal, unperturbed axis of the corresponding element.

### 2.2. Dual-Channel Optical Model

The camera adopts a four-mirror reflective telescope whose primary, secondary, tertiary, and quaternary mirrors (C={1,2,3,4}) are shared by two channels. After the quaternary mirror, the beam diverges: the imaging channel continues through the infrared folding mirror (element 6, the only element of *I*), the infrared lens group, and reaches the infrared focal plane, whereas the measurement channel is routed through the image shift switching mirror (element 7) and the image shift folding mirror (element 8, together constituting M={7,8}) to a lens group and a visible high-frame-rate CMOS with frame rate $f_{s}$. The two channels therefore share the four reflective elements of the telescope and differ only in the three non-shared mirrors indexed 6, 7, and 8; this asymmetry is the source of the recovery rule (1) derived below.

All displacements and angular states are referred to one fixed calibration configuration. Specifically, $\theta_{i}^{\prime }\left(t\right)=\theta_{i}\left(t\right)-\theta_{i,\mathrm{ref}}$ and $P_{k}\left(t\right)=x_{k}\left(t\right)-x_{k,\mathrm{ref}}$, where $x_{k}$ is a detector coordinate in pixels. The reference coordinates and mirror states must correspond to the same optical configuration. Time indices are suppressed below when no ambiguity arises. These quantities are not adjacent-frame increments.

The goal of this section is to establish the recovery rule

(1) $$P_{\mathrm{img}}=P_{\mathrm{meas}}+\frac{\theta_{6}^{\prime }}{\Delta_{6}}+\frac{\theta_{7}^{\prime }}{\Delta_{7}}+\frac{\theta_{8}^{\prime }}{\Delta_{8}},$$

where $\theta_{6}^{\prime }$, $\theta_{7}^{\prime }$, and $\theta_{8}^{\prime }$ are the angular variations of the infrared folding mirror, the image shift switching mirror, and the image shift folding mirror, and $\Delta_{6}$, $\Delta_{7}$, and $\Delta_{8}$ are the corresponding vibration sensitivities. Equation (1) states that the imaging channel shift is obtained by correcting the measurement channel shift with the angular state of the three non-shared mirrors, each weighted by its sensitivity. The remainder of this section derives (1) from the channel equations. This identity requires the non-shared states to be known or estimated from sufficient information; it does not imply that the measurement image contains all three states.

Under the assumptions of Section 2.4, the focal-plane shift in channel *k* is the linear superposition of the contributions of the elements along that channel,

(2) $$P_{k}=\sum_{i\in C\cup M\cup I}S_{i}^{\left(k\right)}\;\theta_{i}^{\prime },\;k\in \left\{\mathrm{img},\mathrm{meas}\right\},$$

where $S_{i}^{\left(k\right)}$ is the image shift sensitivity of element *i* in channel *k* (pixels per radian), with the convention $S_{i}^{\left(k\right)}=0$ when element *i* is not on the path of channel *k*. Physically, a small mirror rotation $\theta_{i}^{\prime }$ changes the reflected chief-ray angle by $2\theta_{i}^{\prime }$. The downstream relay maps this angular perturbation to a focal-plane displacement. In a one-dimensional paraxial representation,

(3) $$\delta x_{i}^{\left(k\right)}=2f_{i,\mathrm{eff}}^{\left(k\right)}\theta_{i}^{\prime },\;\Delta P_{i}^{\left(k\right)}=\frac{\delta x_{i}^{\left(k\right)}}{p}=\frac{2f_{i,\mathrm{eff}}^{\left(k\right)}}{p}\theta_{i}^{\prime },$$

where $f_{i,\mathrm{eff}}^{\left(k\right)}$ is the signed effective angular-to-linear lever arm from mirror *i* to detector *k*, and *p* is the detector pixel pitch. Hence $S_{i}^{\left(k\right)}=2f_{i,\mathrm{eff}}^{\left(k\right)}/p$. In a full ray-tracing model, this scalar is the relevant component of the optical influence matrix; its sign depends on the reflection geometry and detector-axis convention. Equation (3) therefore connects mirror motion, chief-ray deflection, physical focal-plane displacement, and pixel displacement without assuming that every mirror has the same effective lever arm. For the shared front-end elements, the lever arm reduces to the channel focal length, $f_{i,\mathrm{eff}}^{\left(k\right)}=f_{k}$; for the non-shared mirrors, it is instead the mirror-to-detector arm, which generally differs from $f_{k}$. The two are therefore retained as distinct symbols.

Because the shared elements *C* are common to both channels, their vibration-induced contributions are subtracted together in (2). The two channels are designed so that the shared portion (M1–M4) has equal effective focal length, $f_{\mathrm{img}}^{\left(\mathrm{sh}\right)}=f_{\mathrm{meas}}^{\left(\mathrm{sh}\right)}$; a relay/compensator in the measurement branch equalizes the shared-path magnification, so the residual $2\left(f_{\mathrm{img}}^{\left(\mathrm{sh}\right)}-f_{\mathrm{meas}}^{\left(\mathrm{sh}\right)}\right)\theta_{i}/p$ vanishes *exactly* for every shared mirror, for any amplitude $\theta_{i}$. The 27.5% focal length difference quoted between the two channels is confined to their post-split dedicated lens groups and supplies the non-shared-mirror differential sensitivity, so it injects no shared-path residual. The shared terms therefore cancel exactly (Assumption 3) when (2) is subtracted for k=img and k=meas. Defining the correction sensitivity of each non-shared element as

(4) $$\Delta_{i}\;≜\;{\left(S_{i}^{\left(\mathrm{img}\right)}-S_{i}^{\left(\mathrm{meas}\right)}\right)}^{-1},\;i\in \left\{6,7,8\right\},$$

which is obtained off-line by opto-mechanical calibration or ray tracing and absorbs the sign convention of the angles, the difference of the two channel equations becomes

$$P_{\mathrm{img}}-P_{\mathrm{meas}}=\sum_{i\in \{6,7,8\}}\left(S_{i}^{\left(\mathrm{img}\right)}-S_{i}^{\left(\mathrm{meas}\right)}\right)\theta_{i}^{\prime }=\sum_{i\in \{6,7,8\}}\frac{\theta_{i}^{\prime }}{\Delta_{i}},$$

which is exactly (1). Equation (3) and (4) also clarify the physical meaning of the sensitivity $\Delta_{i}$: it scales the angular variation of a non-shared mirror into the pixel-level shift that must be added to or removed from the measurement channel.

For numerical scaling, the synthetic study uses pixel pitch p=5.5μm and nominal channel focal lengths $f_{\mathrm{img}}=1.00\;m$ and $f_{\mathrm{meas}}=0.725\;m$. These two focal lengths alone do not determine the influence coefficient of every mirror. The path-support convention in (2) requires

(5) $$\begin{array}{cccc} S_{6}^{\left(\mathrm{meas}\right)} & =0, & \Delta_{6}^{-1} & =S_{6}^{\left(\mathrm{img}\right)},\\ S_{i}^{\left(\mathrm{img}\right)} & =0, & \Delta_{i}^{-1} & =-S_{i}^{\left(\mathrm{meas}\right)},\;i\in \left\{7,8\right\}, \end{array}$$

In particular, applying $2(f_{\mathrm{img}}-f_{\mathrm{meas}})/p$ to each non-shared mirror would assign a response to a channel that the mirror does not traverse.

The non-shared sensitivities are therefore fixed by the measurement branch geometry rather than chosen freely. Let the two non-shared measurement mirrors be separated by *d* along the unfolded path, let the two observation planes lie at $L_{1}$ and $L_{2}$ downstream of the switching mirror (element 7), and let the imaging-only mirror (element 6) lie $L_{6}$ from the infrared focal plane. With the observation design of Section 5 (d=0.05m, $L_{1}=0.10\;m$, $L_{2}=0.20\;m$, $L_{6}=0.12\;m$), (3) and (5) give

(6) $$\begin{array}{cccc} \frac{1}{\Delta_{6}} & =+\frac{2L_{6}}{p}=+4.36\times 10^{4}\;\mathrm{px}\;{\mathrm{rad}}^{-1},\; & \;\Delta_{6} & =+22.9\;\mu \mathrm{rad}\;{\mathrm{px}}^{-1},\\ \frac{1}{\Delta_{7}} & =-\frac{2L_{1}}{p}=-3.64\times 10^{4}\;\mathrm{px}\;{\mathrm{rad}}^{-1},\; & \;\Delta_{7} & =-27.5\;\mu \mathrm{rad}\;{\mathrm{px}}^{-1},\\ \frac{1}{\Delta_{8}} & =-\frac{2(L_{1}-d)}{p}=-1.82\times 10^{4}\;\mathrm{px}\;{\mathrm{rad}}^{-1},\; & \;\Delta_{8} & =-55.0\;\mu \mathrm{rad}\;{\mathrm{px}}^{-1}, \end{array}$$

and the common (shared-path) motion couples to the measurement image with $C_{\mathrm{sh}}=2f_{\mathrm{meas}}/p=2.64\times 10^{5}\;\mathrm{px}\;{\mathrm{rad}}^{-1}$. The three sensitivities now differ by a factor of 2.4 and carry signs set by the reflection geometry, so they are no longer interchangeable scalars; this is the property that the separate branch layout provides and that the recovery rule (1) depends on. A physical implementation still requires the influence matrix of the actual prescription to be confirmed by ray tracing or experimental calibration; the values above are the paraxial estimates of the stated synthetic geometry.

### 2.3. Observability of the Non-Shared Mirror States

The channel difference identity in (1) is not an observability result. In the stated optical layout, mirror 6 lies exclusively in the imaging branch. For independently variable mirror angles, and with the shared-mirror states, illumination, scene, and detector conditions held fixed, the noiseless measurement image can be written as

(7) $$I_{\mathrm{meas}}=H\left(\theta_{7}^{\prime },\theta_{8}^{\prime };\eta \right),\;\frac{\partial I_{\mathrm{meas}}}{\partial \theta_{6}^{\prime }}=0,$$

where η collects the fixed nuisance variables. This statement uses the separate branch optical model; no mechanical coupling, cross-branch optical interaction, or auxiliary state measurement is included in that model. The same zero derivative applies to any differentiable feature vector $z=\phi (I_{\mathrm{meas}})$, including centroids and PSF moments. Consequently, its three-angle Jacobian has the structure

(8) $$J_{z}=\frac{\partial z}{\partial (\theta_{6}^{\prime },\theta_{7}^{\prime },\theta_{8}^{\prime })}=\left[\begin{array}{ccc} 0 & j_{7} & j_{8} \end{array}\right],\;\mathrm{rank}\left(J_{z}\right)\leq 2.$$

Thus, the smallest of the three singular values is zero, and the three-state inverse problem has no finite full-column-rank condition number. Using more pixels, noise normalization, or a larger regressor does not remove this null direction. Even the rank-two upper bound is not a demonstration that mirrors 7 and 8 are separately observable: that requires independent image signatures and treatment of scene and shared-path nuisance motion.

The missing state also prevents unique recovery of the total imaging channel shift. Consider two states that differ only by a nonzero angle *d* of mirror 6. They produce the same measurement image but, from (1),

(9) $$I_{\mathrm{meas}}^{\left(a\right)}=I_{\mathrm{meas}}^{\left(b\right)},\;P_{\mathrm{img}}^{\left(b\right)}-P_{\mathrm{img}}^{\left(a\right)}=\frac{d}{\Delta_{6}}.$$

Any deterministic measurement image-only estimator returns the same value for these two inputs, so its maximum absolute error over the pair is at least $\left|d|/(2|\Delta_{6}|\right)$. Direct regression of the aggregate shift therefore does not solve the problem either. A learned statistical correlation can predict a hidden state only under the corresponding joint distribution assumptions; it is not the optical identifiability of independently varying angles.

#### 2.3.1. Task-Specific Observability with Nuisance Motion

The required correction is $q=w^{T}\theta$, where $\theta ={\left(\theta_{6}^{\prime },\theta_{7}^{\prime },\theta_{8}^{\prime }\right)}^{T}$ and $w={\left(\Delta_{6}^{-1},\Delta_{7}^{-1},\Delta_{8}^{-1}\right)}^{T}$. Consider the local linear observation model

(10) $$z=Hx+n,\;x={\left(\theta^{T},\nu^{T}\right)}^{T},\;q=\ell^{T}x,\;\ell ={\left(w^{T},0^{T}\right)}^{T},$$

where ν includes unknown shared-path motion and modeled scene or detector nuisance states. All observations use the fixed calibration reference. In this noiseless local model, *q* is uniquely recoverable if and only if

(11) $$\ker H\subseteq \ker\ell^{T},\;\mathrm{equivalently}\;\ell \in \mathrm{range}\left(H^{T}\right).$$

Indeed, indistinguishable states differ by a vector in kerH; their corrections agree precisely when its inner product with *ℓ* vanishes. Equivalently, a vector v must exist such that $H^{T}v=\ell$, giving $q=v^{T}z$ without noise. Recovering the correction can therefore require less information than recovering all angles, but changing the regression output alone cannot remove an unobservable correction direction.

#### 2.3.2. Candidate Additional Observations

A reference design adds synchronized mirror-6 angular metrology, $a_{6}=g_{6}\theta_{6}^{\prime }+n_{6}$, with nonzero calibrated gain $g_{6}$. Its mechanical or optical reference must be stable, independently measured, or included among the nuisance states. Gain, offset, noise, and synchronization require calibration. This is a proposed observation, not an installed sensor. A mechanical constraint can replace it only if independently established.

For mirrors 7 and 8, candidate optical features include spot positions at two observation planes or multiple field points. They help only when the actual propagation geometry yields different sensitivity ratios. After nuisance contributions have been independently determined and removed, write

(12) $$\left[\begin{array}{c} z_{1}\\ z_{2} \end{array}\right]=\underset{B}{\underset{︸}{\left[\begin{array}{cc} b_{17} & b_{18}\\ b_{27} & b_{28} \end{array}\right]}}\left[\begin{array}{c} \theta_{7}^{\prime }\\ \theta_{8}^{\prime } \end{array}\right]+n_{z},\;J_{\mathrm{aug}}=\left[\begin{array}{ccc} g_{6} & 0 & 0\\ 0 & b_{17} & b_{18}\\ 0 & b_{27} & b_{28} \end{array}\right].$$

Three-angle recovery requires $g_{6}\det B\neq 0$. Two copies of the same translated spot do not guarantee this condition. If nuisance motion is unknown, its columns must remain in H; this determinant alone is insufficient. No numerical full-rank matrix is assigned without a supporting optical model.

With only one nuisance-corrected feature $z=b_{7}\theta_{7}^{\prime }+b_{8}\theta_{8}^{\prime }$, the branch correction $q_{78}=\theta_{7}^{\prime }/\Delta_{7}+\theta_{8}^{\prime }/\Delta_{8}$ is recoverable when $\left(\Delta_{7}^{-1},\Delta_{8}^{-1}\right)=\alpha \left(b_{7},b_{8}\right)$ for known α, since $q_{78}=\alpha z$. Otherwise another independent observation is required. Thus $\left(a_{6},z\right)$ is a conditional two-scalar design for correction recovery; the present camera is not assumed to supply a nuisance-free *z*.

#### 2.3.3. Calibration and a Linear Estimation Baseline

Sensitivities must be derived from the selected optical model or controlled calibration. With other states fixed, symmetric perturbations give

(13) $$S_{i}^{\left(k\right)}≃\frac{P_{k}\left(\theta_{0}+he_{i}\right)-P_{k}\left(\theta_{0}-he_{i}\right)}{2h},\;b_{ji}≃\frac{z_{j}\left(\theta_{0}+he_{i}\right)-z_{j}\left(\theta_{0}-he_{i}\right)}{2h}.$$

Here, *h* is in radians; pixel features give sensitivities in px rad^−1^. Signed detector coordinates and $\Delta_{i}^{-1}=S_{i}^{\left(\mathrm{img}\right)}-S_{i}^{\left(\mathrm{meas}\right)}$ must be consistent. Repeating with h/2 and 2h checks local linearity and numerical resolution. Held-out combined perturbations test prediction accuracy. Nuisance-state columns and sensor gains need analogous calibration. Conditioning should use noise whitening and stated state scaling rather than mixed raw angular and pixel units. System focal lengths alone do not specify mirror-specific sensitivities.

For zero-mean observation noise with known covariance R≻0, a minimum-variance linear unbiased correction estimator is

(14) $$v_{*}\in \arg\min_{H^{T}v=\ell }v^{T}Rv,\;\hat{q}=v_{*}^{T}z,\;{\hat{P}}_{\mathrm{img}}={\hat{P}}_{\mathrm{meas}}+\hat{q}.$$

Feasibility is equivalent to (11). This baseline should precede neural comparisons using the same observations. A regressor may address nonlinear or image-dependent effects but cannot create observability; its advantage over this baseline remains to be evaluated. Writing $e_{m}={\hat{P}}_{\mathrm{meas}}-P_{\mathrm{meas}}$, the total variance is $\mathrm{Var}\left(e_{m}\right)+v_{*}^{T}Rv_{*}+2\mathrm{Cov}\left(e_{m},v_{*}^{T}n\right)$. Overlapping image observations must not be assumed independent.

#### 2.3.4. Proposed Stress Settings and Evidence Boundary

The auxiliary-angle noise sweep reported in Section 5.7 applies standard deviations 0.01, 0.05, 0.10, 0.50, and 1.00μrad after gain normalization, together with a signed sensitivity error of up to 10%. These are simulation settings, not instrument specifications. Under the geometry-derived $|\Delta_{6}|=22.9\;\mu \mathrm{rad}\;{\mathrm{px}}^{-1}$, auxiliary noise alone contributes $\sigma_{6}/\left|\Delta_{6}\right|$ pixels RMS, spanning 0.0004–0.044 px, more than an order of magnitude below the network error reported in Section 5. A small time offset τ contributes approximately $|{\dot{\theta }}_{6}^{\prime }\tau /\Delta_{6}|$ pixels, requiring a disturbance-dependent synchronization budget. Reference bias and gain error must be assessed separately from zero-mean noise.

#### 2.3.5. Structural Scale of the Observable Correction

Because the null direction of (8) is unobserved, the common-path motion can be removed only to the accuracy of the independent reference used to compensate it. Writing δμ for the compensation residual at the measurement plane, the states that are consistent with a given measurement patch differ in $q_{78}$ by exactly $C_{\mathrm{sh}}\delta \mu$. The conditional uncertainty of the correction given the patch is therefore governed by that quantity,

(15) $$\mathrm{std}\left(q_{78}|I_{\mathrm{meas}}\right)=C_{\mathrm{sh}}\;\mathrm{std}\left(\delta \mu |I_{\mathrm{meas}}\right)\;\leq \;C_{\mathrm{sh}}\;\sigma_{\delta \mu },\;\sigma_{\delta \mu }=\mathrm{std}\left(\delta \mu \right),$$

which sets the structural scale of the correction: the scale is approached when the angular prior is diffuse and can only be tightened by a bounded mirror-angle envelope. For the nominal residual $\sigma_{\delta \mu }=0.5\;\mu$rad it is 0.132 px. Section 5 shows that the trained estimator attains it (0.130 px): beyond this point, the correction accuracy is set by the common-path reference rather than by the network capacity, and a more accurate common-path reference is the only way to lower it.

This augmented design is a conditional implementation specification. The numerical study of Section 5 and Section 5.7 evaluates the observable correction $q_{78}$ with the auxiliary mirror-6 term supplied analytically under a stated noise model; it does not synthesize the auxiliary metrology spots as images, and it does not validate the physical forward model or the information paths of the stated prescription.

### 2.4. Assumptions

The model above relies on the following assumptions.

**Assumption** **1.** *The angular variations $\theta_{i}^{\prime }$ are small, of the order of microradians, so that the reflected-ray deflection is well approximated by $2\theta_{i}^{\prime }$ and the focal-plane shift is linear in $\theta_{i}^{\prime }$.*

**Assumption** **2.** *The image shift sensitivities $S_{i}^{\left(k\right)}$ and the correction sensitivities $\Delta_{i}$ are constant over the operating range of the camera. They are established by opto-mechanical calibration or ray tracing and remain valid as long as the structural configuration does not change.*

**Assumption** **3.** *The shared telescope (M1–M4) is designed so that the two channels have equal effective focal length over the shared path (a relay/compensator in the measurement branch equalizes the shared-path magnification). The shared contribution to the difference (1) is therefore $2\left(f_{\mathrm{img}}^{\left(\mathrm{sh}\right)}-f_{\mathrm{meas}}^{\left(\mathrm{sh}\right)}\right)\theta_{i}/p=0$ exactly, for any shared-mirror motion $\theta_{i}$, so the shared terms cancel regardless of whether the shared mirrors vibrate. The 27.5% focal length difference is confined to the post-split dedicated lens groups and supplies the non-shared-mirror differential sensitivity, not a shared-path residual. Any residual channel imbalance is absorbed by the calibrated sensitivities $\Delta_{i}$.*

Assumptions 1 and 2 are standard in opto-mechanical vibration analysis [21,45] and are revisited in Section 4, where their violation is discussed.

## 3. Proposed Hybrid Method

### 3.1. Overall Pipeline

The proposed method is designed to estimate the imaging channel image shift without a full structural re-solve during inference and without interrupting normal imaging. Its numerical evaluation is simulation-based; the timing reported below is a compute-only estimate rather than measured end-to-end hardware latency. The dual-channel layout of Section 2 provides at every instant a measurement channel frame $I_{t}$ captured by the visible CMOS at frame rate $f_{s}=2000\;\mathrm{Hz}$ (Table 1), while the imaging channel keeps operating undisturbed and its shift is reconstructed rather than measured. The following four-stage computation is the estimator evaluated in Section 5. It regresses only the *observable* part of the correction and requires the auxiliary mirror-6 metrology identified in Section 2.3; that auxiliary term is modeled analytically under a stated noise model rather than synthesized as an image.

First, the centroid $c_{t}$ of the imaged target (a focal-plane reticle or scene feature) is tracked in detector coordinates in $I_{t}$. With a fixed calibrated reference centroid $c_{\mathrm{ref}}$, the measured displacement is

(16) $${\hat{P}}_{\mathrm{meas}}\left(t\right)=c_{t}-c_{\mathrm{ref}},$$

in pixels. The reference is not replaced by the previous-frame centroid. When a moving crop is used, its detector coordinate origin must be included in $c_{t}$; crop-centered coordinates alone discard the target translation. The angular labels and estimates use the same calibration reference. For a scene feature, this model assumes that intrinsic target motion is negligible or has been accounted for separately. Second, a short buffer of centroid-shift samples may be Fourier-transformed to reveal the dominant disturbance frequency; this diagnostic validates the vibration band off-line but does not enter the shift estimate. Third, a lightweight quantized neural network N ingests the single measurement frame $I_{t}$ and produces one scalar, the observable part of the correction,

(17) $${\hat{q}}_{78}=N\left(I_{t}\right),\;q_{78}≜\frac{\theta_{7}^{\prime }}{\Delta_{7}}+\frac{\theta_{8}^{\prime }}{\Delta_{8}}.$$

**Figure 1:**
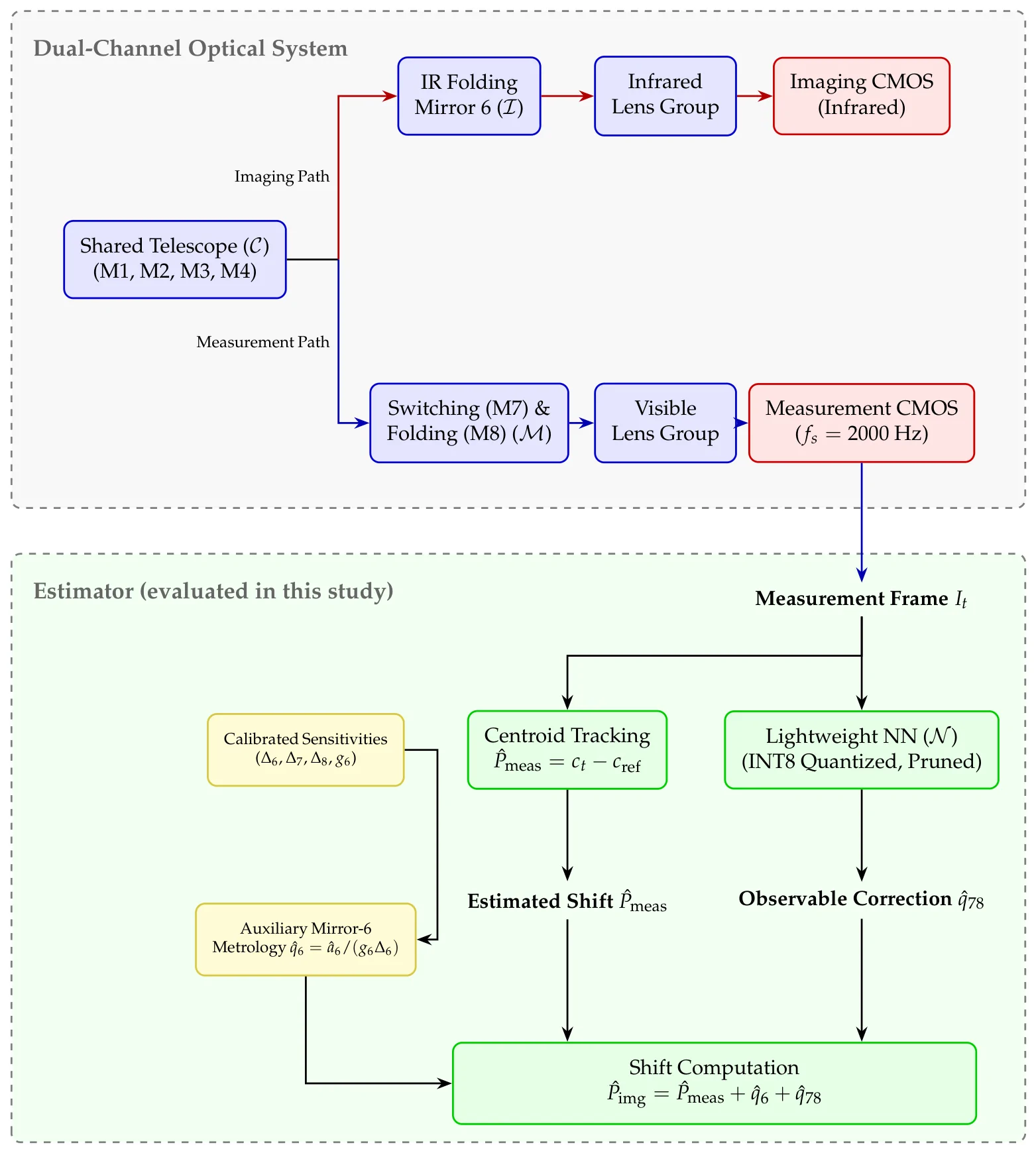
Block diagram of the dual-channel measurement concept and of the estimator evaluated in this paper. The boxes denote functional stages; they are not drawn to optical scale, and the figure shows no optical element geometry, ray paths, or beam-splitting optics. **Top**: after the shared primary elements (M1–M4) the beam is divided into an infrared imaging branch (mirror 6 and the infrared lens group) and a visible measurement branch (mirrors 7 and 8 and the visible lens group). **Bottom**: the estimator evaluated in this paper. Because mirror 6 has no optical path to the measurement CMOS, the network regresses only the observable correction $q_{78}$ from the measurement frame, and the mirror-6 term $q_{6}$ is supplied by the auxiliary angular metrology discussed in Section 2.3; that auxiliary observation is modeled analytically and is not an installed sensor. The diagram does not represent a constructed laboratory instrument or a demonstrated compensation loop.

The network is not asked to output $\theta_{6}^{\prime }$, because Section 2.3 shows that mirror 6 leaves no signature in $I_{t}$; a network that reported it would be returning an unobservable state. The mirror-6 contribution is instead supplied by the auxiliary angular metrology of Section 2.3 as ${\hat{q}}_{6}={\hat{a}}_{6}/\left(g_{6}\Delta_{6}\right)$, where $g_{6}$ is the calibrated metrology gain. Fourth, the two terms are added to the measured channel shift,

(18) $$\hat{P_{\mathrm{img}}}=\hat{P_{\mathrm{meas}}}+{\hat{q}}_{6}+{\hat{q}}_{78},$$

which is (1) with the sum over non-shared elements grouped into the observable part $q_{78}$ and the auxiliarily observed part $q_{6}$. In the simulated pipeline, $\hat{P_{\mathrm{meas}}}$ and $I_{t}$ are available at every CMOS frame and one estimate $\hat{P_{\mathrm{img}}}$ is computed per network forward pass. Whether a physical implementation sustains the full $f_{s}$ rate also depends on sensor exposure and readout, data transfer, synchronization, centroid tracking, and post-processing, none of which is included in the present compute-only timing estimate. Section 3.2.2 gives an order-of-magnitude allocation of the 0.5 ms frame interval across these non-inference stages. Figure 1 summarizes the proposed dual-channel optics and processing chain.

For a physical implementation, the centroid estimate must be quality-gated before it enters (18). A frame is marked invalid when the target contrast or valid-pixel fraction falls below a calibrated threshold, when the centroid is saturated or absent, or when a frame timestamp is missing. In that case, the pipeline must flag the shift estimate as unavailable rather than interpret the failed centroid as physical jitter. A controller may hold the last valid correction for a bounded interval, propagate a filtered state with increased uncertainty, and require re-acquisition before resuming updates. Algorithm 1 formalizes this policy as a quality gate followed by a bounded hold: a frame that fails the gate is either held, for at most *H* consecutive frames, or flagged invalid until re-acquisition, and no temporal predictor is applied. Cosmic-ray rejection, redundant features, and a Kalman-style predictor are candidate implementations extensions; propagating a filtered state with increased uncertainty is not evaluated here. Section 5.9 evaluates a simpler flux/saturation gate and bounded-hold policy for an explicit linear auxiliary-measurement baseline; Kalman prediction and robustness of the original neural estimator remain untested.
**Algorithm 1** Image shift estimator: observable correction from the measurement image plus auxiliary mirror-6 metrology, with quality gating and bounded hold.**Require:** calibrated sensitivities $\Delta_{6},\Delta_{7},\Delta_{8}$ and metrology gain $g_{6}$ (off-line, Equations (4) and (6)); INT8 network N; measurement-frame stream $I_{t}$ at rate $f_{s}$; synchronized mirror-6 reading $a_{6}$; fixed reference centroid $c_{\mathrm{ref}}$; quality-gate thresholds (contrast, valid-pixel fraction, saturation, and timestamp completeness) and a maximum hold *H***Ensure:** imaging channel image shift $\hat{P_{\mathrm{img}}}$ per frame, or an invalid flag {auxiliary observation modeled as synchronous} 1:initialize N, $\Delta_{6},\Delta_{7},\Delta_{8}$, $g_{6}$; load the calibrated centroid $c_{\mathrm{ref}}$ in detector coordinates 2:$n_{\mathrm{hold}}\leftarrow 0$; ${\hat{P_{\mathrm{img}}}}^{\mathrm{last}}\leftarrow \mathrm{NONE}$ {no valid estimate yet} 3:**for** each incoming frame $I_{t}$ **do** 4:   **if** QualityGate($I_{t}$) is valid **then** 5:     track the target centroid $c_{t}$ in $I_{t}$ 6:     $\hat{P_{\mathrm{meas}}}\leftarrow c_{t}-c_{\mathrm{ref}}$ {measurement channel shift, Equation (16)} 7:     ${\hat{q}}_{78}\leftarrow N\left(I_{t}\right)$ {observable correction, Equation (17)} 8:     ${\hat{q}}_{6}\leftarrow a_{6}/\left(g_{6}\Delta_{6}\right)$ {auxiliary mirror-6 term} 9:     ${\hat{P_{\mathrm{img}}}}^{\mathrm{last}}\leftarrow \hat{P_{\mathrm{meas}}}+{\hat{q}}_{6}+{\hat{q}}_{78}$ {recovery rule, Equation (1)}10:     $n_{\mathrm{hold}}\leftarrow 0$11:     output ${\hat{P_{\mathrm{img}}}}^{\mathrm{last}}$12:   **else if** ${\hat{P_{\mathrm{img}}}}^{\mathrm{last}}\neq \mathrm{NONE}$ and $n_{\mathrm{hold}}<H$ **then**13:     $n_{\mathrm{hold}}\leftarrow n_{\mathrm{hold}}+1$14:     output ${\hat{P_{\mathrm{img}}}}^{\mathrm{last}}$ {held; flagged as held, not as a new estimate}15:   **else**16:     output invalid {re-acquisition required before resuming}17:   **end if**18:**end for**

If adjacent-frame motion is needed as a separate output, it is obtained by differencing the reference-relative estimates. For fixed sensitivities,

(19) $$\delta P_{\mathrm{img}}\left(t\right)=\delta P_{\mathrm{meas}}\left(t\right)+\sum_{i\in \{6,7,8\}}\frac{\theta_{i}^{\prime }\left(t\right)-\theta_{i}^{\prime }\left(t-1\right)}{\Delta_{i}},\;\delta P_{k}\left(t\right)=P_{k}\left(t\right)-P_{k}\left(t-1\right).$$

Thus, a centroid increment must be combined with angle increments, rather than with the current reference-relative angles. The main estimator and reported displacement metric use the fixed-reference convention.

### 3.2. Lightweight Quantized Network

The regressor N is designed for on-board execution under tight memory and compute budgets, in contrast to a full FEA model that must be re-solved whenever the structural configuration changes [9,10].

#### 3.2.1. Input, Backbone, and Outputs

The input is a single grayscale measurement-frame patch $I_{t}\in R^{64\times 64}$ centered on the tracked target; the patch is read out in detector coordinates so that it contains the target translation rather than discarding it. No temporal stacking is required, so the network requires one forward pass per frame; acquisition, tracking, and transfer costs are additional. A shallow convolutional backbone whose stages are depthwise-separable convolutions (the inverted-residual building block of MobileNetV2 [42]) extracts features, which are reduced by global average pooling and mapped by two fully connected layers to the *one-dimensional* output $N\left(I_{t}\right)\in R^{1}$ corresponding to the observable correction $q_{78}$ of (17). The exact layout and hyperparameters are summarized in Table 2. Five separable stages with increasing width (32–128 channels) halve the spatial resolution at each stride-2 step, so the feature map is compressed from $64^{2}$ to $2^{2}$ before the pooling and regression head. The full-precision network has 34,171 parameters; with 80% of the output channels retained per stage after structured pruning (Section 3.2.3) it shrinks to 23,614, well within the memory envelope of a spacecraft processor [37,43].

#### 3.2.2. Why This Lightweight Backbone

The choice of a depthwise-separable stack is dictated by the 2000Hz on-board budget rather than by accuracy alone. A standard convolution of kernel *K* mixing $C_{\mathrm{in}}$ into $C_{\mathrm{out}}$ channels costs $K^{2}C_{\mathrm{in}}C_{\mathrm{out}}$ multiply–accumulates per spatial position, whereas the separable form splits this into a depthwise term $K^{2}C_{\mathrm{in}}$ and a pointwise term $C_{\mathrm{in}}C_{\mathrm{out}}$, a reduction of roughly $K^{2}/C_{\mathrm{out}}$ for the early wide stages. For the present input the full-precision cost is $9.02\times 10^{5}$ multiply–accumulates per frame ($9.0\times 10^{-4}$ G), and its distribution is not what a purely spatial argument would suggest: the $16^{2}$ stage dominates with 51.8% of the total, whereas the $64^{2}$ input stage contributes only 4.7%, because the depthwise term scales with $C_{\mathrm{in}}$ while the pointwise term scales with $C_{\mathrm{in}}C_{\mathrm{out}}$. Retaining 80% of the channels per stage lowers the cost to $6.1\times 10^{-4}$ G. Counting one multiply–accumulate as two operations and assuming the 120 GOPS INT8 throughput used throughout this study, the projected network-compute time is 0.0150 ms per frame, about 3% of the 0.5 ms frame interval; the same convention gives 0.0101 ms for the pruned variant. This is a compute-only budget, not a measured latency: memory traffic, scheduling, synchronization, and the remaining pipeline stages are excluded, and a network of this size on an embedded target is frequently memory-bound rather than MAC-bound. Global average pooling replaces a large fully connected front-end with a single reduction, and the two-layer head is the minimal regressor for a one-dimensional continuous output. We deliberately forgo the inverted-residual expansion factor of MobileNetV2: the unexpanded separable stage is sufficient for the smooth, low-dimensional angle manifold and preserves the smallest footprint. The narrow, shallow architecture is intended to limit inference cost. Any future on-board retraining would require a separate assessment of training data, memory, energy, and execution time; it is not evaluated here. This design choice is not presented as a proof of global architectural optimality: no controlled comparison against alternative backbones is reported. It is selected as a transparent low-parameter baseline for the synthetic regression study, with its parameter count, MAC count, pruning convention, and limits of the projected compute estimate reported explicitly.

As shown in Table 3, the estimated digital chain uses about one tenth of the 0.5 ms interval. With a slower 100 MB/s link, its subtotal rises to about 70 μs (14%), leaving about 430 μs for exposure, readout, and other uncounted stages. The exposure/readout row gives the residual time available within the frame interval, not an estimated or measured duration. DMA transfer is the largest of the listed non-inference digital terms. End-to-end operation at 2000 Hz requires the entire acquisition and processing chain to meet the 0.5 ms deadline.

#### 3.2.3. Loss and Quantization

The network is trained with the mean squared error on the observable correction,

(20) $$L=\frac{1}{N}\sum_{n=1}^{N}{\left(q_{78}^{\left(n\right)}-N\left(I_{t}^{\left(n\right)}\right)\right)}^{2},$$

where $q_{78}^{\left(n\right)}$ is the ground-truth correction of sample *n* (17). The loss is minimized with the Adam optimizer (initial learning rate $10^{-3}$, cosine decay over 30 epochs, batch size 256); no weight decay is used because the network is heavily regularized by its small capacity.

After training, the network is compressed in two steps [39,40,41]. First, *structured pruning* removes entire output channels per stage, ranked by the $\ell_{2}$ norm of the corresponding pointwise filter. The measured accuracy–size trade-off is reported in Section 5.8: retaining 80% of the channels per stage reduces the parameter count from 34,171 to 23,614 and the MAC count from $9.0\times 10^{-4}$ to $6.1\times 10^{-4}$ G at a cost of 0.014 px of RMSE, whereas a more aggressive 40% setting degrades the RMSE to 0.605 px and is not usable. Second, *post-training INT8 quantization* maps every weight and activation to a signed 8-bit integer through a per-layer affine transform $\hat{x}=\mathrm{clip}(\mathrm{round}\left(x/s+z\right),-$128,127), where the scale *s* and zero-point *z* are found by minimizing the quantization error on a small 256-sample calibration set drawn from the training distribution, and clip saturates overflows. Biases are kept at INT32 accumulators so that the dot products do not lose dynamic range, and the rounding is unbiased (stochastic during calibration, nearest during quantized inference). Because the network output is a single scalar with a narrow dynamic range, one per-tensor scale is sufficient for both weights and activations: no per-channel weight scaling is needed, which keeps the inference kernel a pure integer multiply–accumulate. Measured on the held-out test set, INT8 raises the correction RMSE from 0.130 to 0.133 px (+3%) and INT4 collapses to 0.757 px; INT8 is therefore retained (Section 5.8). The combination of pruning and INT8 reduces the model footprint from 0.137 MB to 0.024 MB while preserving the regression accuracy needed for sub-pixel shift recovery (Section 5). The INT8 network is the candidate for a future on-board implementation; the floating-point variant serves as an offline reference. Neither variant has been evaluated on flight hardware in this study.

### 3.3. Training and Inference Protocol

The angular states of the three non-shared mirrors are drawn independently and uniformly from [−80,80]μrad [3,6,7]; this envelope spans the microradian-to-sub-mrad range of spacecraft micro-vibration and stays well inside the small-angle regime quantified in Section 4.2. Sampling the envelope uniformly, rather than from one particular disturbance spectrum, makes the study insensitive to the assumed band structure and gives the ground-truth correction $q_{78}=\theta_{7}^{\prime }/\Delta_{7}+\theta_{8}^{\prime }/\Delta_{8}$ a standard deviation of 1.87 px. The common-path motion is compensated in the front end up to a residual $\delta \mu ∼N(0,\sigma_{\delta \mu }^{2})$ with $\sigma_{\delta \mu }=0.5\;\mu$rad, which enters the measurement image through $C_{\mathrm{sh}}$ and sets the structural scale (15).

Each sample is one rendered 64×64 measurement patch, generated by the measurement forward model: the two observation planes receive Gaussian spots at ±16 px whose displacements follow $z_{k}=A_{k7}\theta_{7}^{\prime }+A_{k8}\theta_{8}^{\prime }+C_{\mathrm{sh}}\delta \mu$; the detector then applies fixed photoresponse non-uniformity (PRNU, 1.0% RMS gain) and dark-signal non-uniformity (DSNU, 0.5% RMS offset), a per-sample background gradient (3% linear, 1% quadratic), read noise (σ=0.01 of full scale), and signal-dependent shot noise ($0.005\sqrt{I}$), clipped to [0,1].

Training uses 20,000 patches per epoch drawn on-line from this model, with a fixed validation set of 5000 and a fixed test set of 10,000 patches. The three sets share the same calibrated detector realization but no sample, and the test set is never used during training or model selection. Training is performed off-line for 30 epochs using the schedule of Section 3.2.3; on an RTX 5060 Laptop GPU it converges in about 4 min with the training and validation curves tracking each other, so no overfitting is observed. At inference, each CMOS frame $I_{t}$ is processed by the pruned INT8 network and Algorithm 1. Recalibration of $\Delta_{i}$ and the metrology gain $g_{6}$ changes only the two closed-form terms ${\hat{q}}_{6}$ and ${\hat{q}}_{78}$.

Band-limited behavior is checked separately, without retraining, on trajectories whose mirror angles are sums of harmonics drawn from the 48–52, 115–125, and 190–210 Hz bands and on deliberately unseen bands (Section 5.4).

Algorithm 1 applies a single quality gate before the recovery rule of Equation (18). A frame that fails the gate is not silently interpreted as physical jitter. If no valid estimate is available, it is flagged invalid immediately; otherwise, the last valid estimate is held for at most *H* consecutive frames and then reported as invalid until re-acquisition. No temporal predictor is applied inside the loop. The hold bound and the gate thresholds are simulation settings rather than flight-qualified values; the explicit auxiliary baseline of Section 5.9 uses H=3 and a flux/saturation gate, whereas a Kalman-style predictor remains an untested extension (Section 4.2).

## 4. Analysis and Discussion

This section derives an end-to-end error budget for the imaging channel shift estimate and discusses its assumptions and the limits of the simulation evidence. An estimation error budget does not establish a processing deadline or hardware feasibility.

### 4.1. Error Propagation

The decomposition below is exact given the estimator structure, and the accuracy of the correction estimate is additionally limited by the structural scale (15). All numerical values quoted here are measured on the simulated data of Section 5, not assumed. Let $\hat{P_{\mathrm{img}}}$ be the estimate produced by Algorithm 1. Substituting the estimated terms into (18) and subtracting the true shift $P_{\mathrm{img}}$ defined by (1) gives the estimation error

(21) $$e\;=\;P_{\mathrm{img}}-\hat{P_{\mathrm{img}}}\;=\;\underset{\varepsilon_{c}}{\underset{︸}{P_{\mathrm{meas}}-\hat{P_{\mathrm{meas}}}}}\;+\;\underset{\varepsilon_{6}^{P}}{\underset{︸}{\frac{\theta_{6}^{\prime }-\hat{\theta_{6}^{\prime }}}{\Delta_{6}}}}\;+\;\underset{\varepsilon_{78}}{\underset{︸}{q_{78}-{\hat{q}}_{78}}},$$

where $\hat{P_{\mathrm{meas}}}$ is the centroid-based measurement channel shift, $\hat{\theta_{6}^{\prime }}$ the auxiliary mirror-6 estimate, and ${\hat{q}}_{78}$ the network output. The first term is the centroid tracking error relative to the fixed reference; the second propagates the auxiliary metrology error; the third is the correction regression error. The angle errors of mirrors 7 and 8 do not appear separately, because the network regresses their combined contribution $q_{78}$ and only the error of that combination enters the shift. Thus, $\sigma_{c}$ describes displacement estimation noise, not automatically the noise of one raw centroid. If the current and reference centroid errors are $n_{t}$ and $n_{\mathrm{ref}}$, then $\varepsilon_{c}=n_{\mathrm{ref}}-n_{t}$ and

(22) $$\mathrm{Var}\left(\varepsilon_{c}\right)=\mathrm{Var}\left(n_{t}\right)+\mathrm{Var}\left(n_{\mathrm{ref}}\right)-2\mathrm{Cov}\left(n_{t},n_{\mathrm{ref}}\right).$$

A fixed reference error can also introduce a trajectory-wide bias and cross-time correlation. The zero-mean bounds below require this bias to be removed or accounted for; they do not imply temporal independence.

**Lemma** **1** (Error decomposition)**.** *Under Assumptions 1–3, if $\varepsilon_{c}$, $\varepsilon_{6}^{P}$ and $\varepsilon_{78}$ are zero-mean and mutually independent, the mean squared estimation error is*

(23) $$E\left[e^{2}\right]=\sigma_{c}^{2}+{\left(\frac{\sigma_{6}}{\Delta_{6}}\right)}^{\;2}+\mathrm{MSE}\left(q_{78}\right),$$

*with $\sigma_{c}=\mathrm{std}\left(\varepsilon_{c}\right)$, $\sigma_{6}$ the standard deviation of the mirror-6 metrology error, and $\mathrm{MSE}\left(q_{78}\right)=E\left[{\left(q_{78}-{\hat{q}}_{78}\right)}^{2}\right]$.*

**Proof.**  Write $e=\varepsilon_{c}+\varepsilon_{6}^{P}+\varepsilon_{78}$ as in (21). By hypothesis, the three terms are mutually independent and zero mean, so $E\left[e^{2}\right]=\mathrm{Var}\left(e\right)=\mathrm{Var}\left(\varepsilon_{c}\right)+\mathrm{Var}\left(\varepsilon_{6}^{P}\right)+\mathrm{Var}\left(\varepsilon_{78}\right)$, and $\mathrm{Var}\left(\varepsilon_{6}^{P}\right)=\mathrm{Var}\left(\varepsilon_{6}\right)/\Delta_{6}^{2}$ because $\varepsilon_{6}^{P}=\varepsilon_{6}/\Delta_{6}$. □

The independence condition in Lemma 1 is an analytical simplification and not an experimentally verified property of the proposed system. If the centroid and propagated angle errors are correlated, the exact mean squared error additionally contains the cross-covariance terms $2\sum_{a<b}\mathrm{Cov}\left(\varepsilon_{a},\varepsilon_{b}\right)$ for $\varepsilon_{a},\varepsilon_{b}\in \left\{\varepsilon_{c},\varepsilon_{6}^{P},\varepsilon_{7}^{P},\varepsilon_{8}^{P}\right\}$. The diagonal expression in (23) and the bounds derived from it should therefore be read as conditional model results. A physical implementation must estimate or bound these covariances from held-out synchronized measurements before using the decomposition as an uncertainty budget.

**Theorem** **1** (Correction scale and end-to-end budget)**.** *Let the auxiliary metrology error be zero-mean with standard deviation $\sigma_{6}$, and let the correction be estimated from the measurement image under a common-path compensation residual $\sigma_{\delta \mu }$. Then, (i) the conditional standard deviation of $q_{78}$ given the measurement patch is bounded by the structural scale $C_{\mathrm{sh}}\sigma_{\delta \mu }$ of (15), so the Bayes-optimal image-based correction error cannot exceed that scale, and (ii) under the independence approximation of Lemma 1, the mean squared end-to-end error of Algorithm 1 is*

(24) $$E\left[e^{2}\right]=\sigma_{c}^{2}+\frac{\sigma_{6}^{2}}{\Delta_{6}^{2}}+\mathrm{MSE}\left(q_{78}\right),\;\mathrm{MSE}\left(q_{78}\right)\;≲\;{\left(C_{\mathrm{sh}}\;\sigma_{\delta \mu }\right)}^{2},$$

*so the end-to-end error is the quadrature sum of the displacement tracking error, the auxiliary metrology error, and the correction scale. The scale is not a performance guarantee for the network: it marks the level at which the common-path reference, rather than the regressor, becomes the limiting term.*

**Proof.**  For (i), the states consistent with a noiseless patch form a one-parameter family parameterized by δμ, along which $q_{78}$ changes by exactly $C_{\mathrm{sh}}\delta \mu$; hence $\mathrm{std}\left(q_{78}|I_{\mathrm{meas}}\right)=C_{\mathrm{sh}}\;\mathrm{std}\left(\delta \mu |I_{\mathrm{meas}}\right)\leq C_{\mathrm{sh}}\sigma_{\delta \mu }$, which is (15). For (ii), Lemma 1 gives $E\left[e^{2}\right]=\sigma_{c}^{2}+{\left(\sigma_{6}/\Delta_{6}\right)}^{2}+\mathrm{MSE}\left(q_{78}\right)$, and the correction term is bounded by the scale of (i). □

For the operating point of this study the three contributions are measured rather than assumed: the centroid displacement error is $\sigma_{c}=0.378\;\mathrm{px}$ (the localization error of the threshold-weighted estimator of Section 5.2 on the simulated frames), the auxiliary term is $\sigma_{6}/\Delta_{6}=0.002\;\mathrm{px}$ at $\sigma_{6}=0.05\;\mu$rad, and the correction term reaches the structural scale $C_{\mathrm{sh}}\sigma_{\delta \mu }=0.132\;\mathrm{px}$. Their quadrature sum is 0.400px, and the end-to-end RMSE obtained by substituting the trained network into (18) is 0.398px, so the budget accounts for the measured error to within 1%. The breakdown also identifies the bottleneck: of the 0.398px, the centroid displacement term contributes 0.378px, whereas the correction regressor contributes only 0.130px and the auxiliary metrology 0.002px. Lowering the end-to-end error therefore requires a better displacement measurement and a more accurate common-path reference, not a larger network. For reference, ignoring the non-shared mirrors entirely and reporting $\hat{P_{\mathrm{meas}}}$ as the imaging shift gives 2.62px; the same quadrature over-estimates that estimator (2.78px) because its error terms are correlated, which is the caveat attached to Lemma 1 and quantified in Section 5.3.

#### 4.1.1. Linearization Error

The exact focal-plane displacement produced by rotating a flat mirror through $\theta_{i}$ is $d_{i}^{\left(k\right)}=\left(f_{i,\mathrm{eff}}^{\left(k\right)}/p\right)\tan\left(2\theta_{i}\right)$ (the reflected ray is deflected by exactly $2\theta_{i}$), whereas (3) approximates it by $\left(2f_{i,\mathrm{eff}}^{\left(k\right)}/p\right)\theta_{i}$. The per-mirror linearization remainder in channel *k* is therefore

(25) $$r_{i}^{\left(k\right)}=\frac{f_{i,\mathrm{eff}}^{\left(k\right)}}{p}\left[\tan\left(2\theta_{i}\right)-2\theta_{i}\right].$$

In the recovered shift (1) the shared elements contribute only a negligible residual (Assumption 3), so to leading order only the three non-shared mirrors remain, each weighted by $S_{i}^{\left(\mathrm{img}\right)}-S_{i}^{\left(\mathrm{meas}\right)}=1/\Delta_{i}$; the total linearization error of the estimate is

(26) $$e_{\mathrm{lin}}=\sum_{i\in \{6,7,8\}}\frac{1}{2\Delta_{i}}\left[\tan\left(2\theta_{i}\right)-2\theta_{i}\right].$$

**Lemma** **2** (Linearization error bound)**.** *Under Assumption 1 with $|\theta_{i}|\leq \theta_{\max}$ and $\theta_{\max}\ll 1$,*

(27) $$|e_{\mathrm{lin}}|\leq \frac{3}{2\Delta_{\min}}\left[\tan\left(2\theta_{\max}\right)-2\theta_{\max}\right].$$

**Proof.**  The function g(x)=tanx−x is non-negative and strictly increasing on [0,π/2); thus $|\tan\left(2\theta_{i}\right)-2\theta_{i}|\leq \tan\left(2\theta_{\max}\right)-2\theta_{\max}$, and (26) with $|\Delta_{i}|\geq \Delta_{\min}$ gives (27). □

For the geometry-derived $\Delta_{\min}=\min_{i}\left|\Delta_{i}\right|=22.9\;\mu \mathrm{rad}\;{\mathrm{px}}^{-1}$ and the simulated envelope $\theta_{\max}=80\;\mu \mathrm{rad}$ (Section 5); with $\tan x-x=x^{3}/3+O\left(x^{5}\right)$, $\tan\left(2\theta_{\max}\right)-2\theta_{\max}\approx 8\theta_{\max}^{3}/3\approx 1.4\times 10^{-12}\;\mathrm{rad}$, so $|e_{\mathrm{lin}}|\leq 8.9\times 10^{-8}\;\mathrm{px}$. This is six orders of magnitude below the centroid displacement error $\sigma_{c}=0.378\;\mathrm{px}$, so Assumption 1 introduces no meaningful error anywhere in the simulated envelope and the linear model of Section 2 is self-consistent over this operating range.

#### 4.1.2. Quantization-Aware Refinement

Let the full-precision network achieve a correction mean squared error ${\mathrm{MSE}}_{\mathrm{fp}}\left(q_{78}\right)$, and let INT8 quantization inject an additional zero-mean error of standard deviation $\delta_{q}$ that is uncorrelated with the full-precision error. The quantized estimator then has

(28) $${\mathrm{MSE}}_{q}\left(q_{78}\right)={\mathrm{MSE}}_{\mathrm{fp}}\left(q_{78}\right)+\delta_{q}^{2}.$$

Inserting (28) into Lemma 1 raises the network term of (24) by $\delta_{q}^{2}$. On the held-out test set, the measured correction RMSE grows from 0.1298px (full precision) to 0.1334px (INT8), so $\delta_{q}=\sqrt{0.1334^{2}-0.1298^{2}}=0.031\;\mathrm{px}$ and the end-to-end RMSE of (24) moves from 0.398 to 0.401px. For a scalar output with a narrow dynamic range the price of INT8 is therefore 0.03px of correction error, two orders of magnitude below the centroid displacement term. Equation (28) describes a modeled precision–accuracy trade-off; it does not verify the resource usage or timing of a hardware implementation.

**Figure 2:**
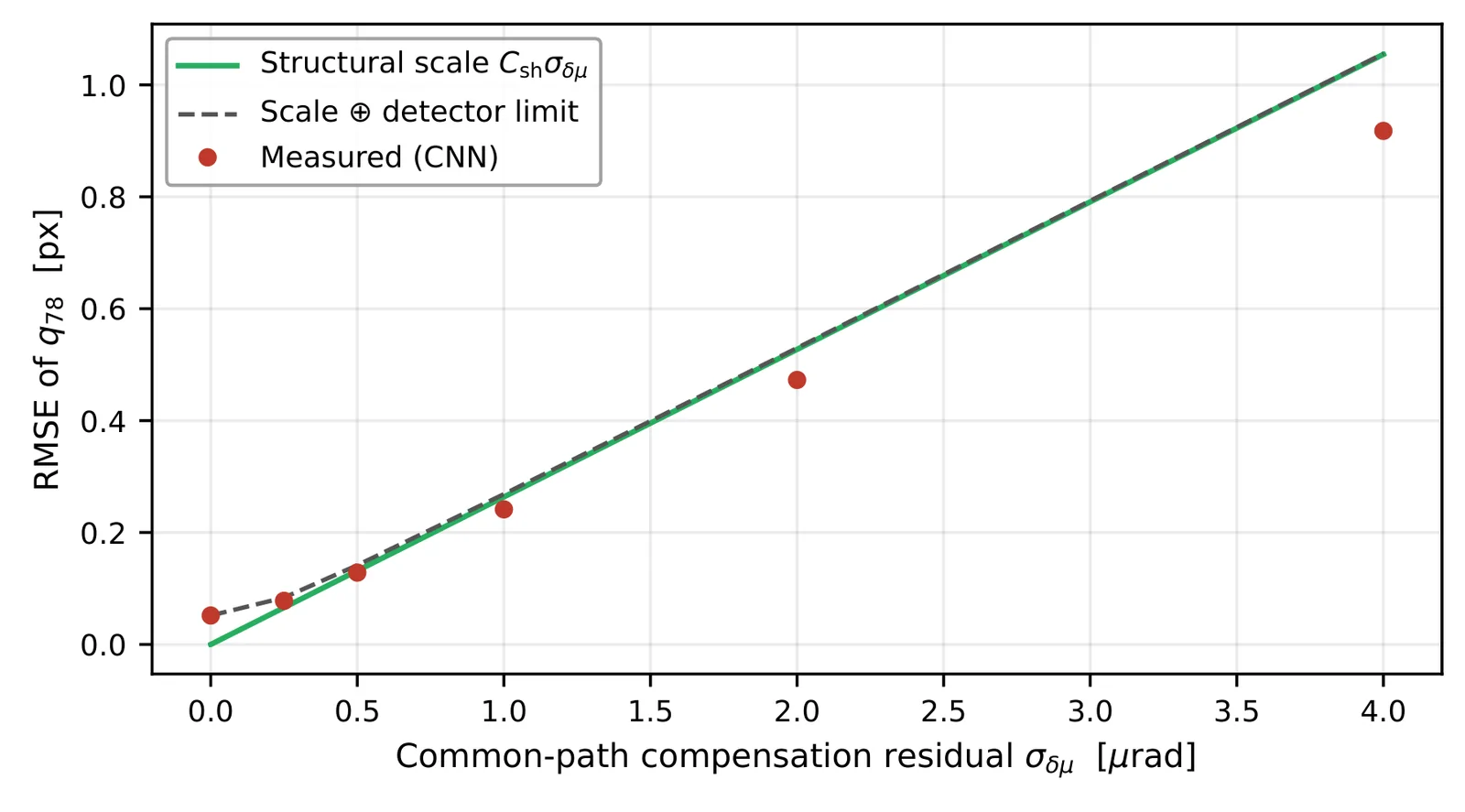
Structural scale of the observable correction. The solid line is $C_{\mathrm{sh}}\sigma_{\delta \mu }$ from (15), the dashed line adds the detector noise limit of the regression in quadrature, and the markers are the correction RMSE measured with the trained network as the common-path compensation residual is swept. The measured curve follows the structural scale, so the correction accuracy is set by the common-path reference rather than by the regressor.

Figure 2 plots the structural scale $C_{\mathrm{sh}}\sigma_{\delta \mu }$ and the same scale combined in quadrature with the detector noise limit of the regression against the measured correction RMSE as $\sigma_{\delta \mu }$ is swept over two decades. At zero residual, the measured error is 0.052 px, which is the detector noise limit of the regression; it then follows the structural scale, reaching 0.128 px at the nominal 0.5μrad and 0.917 px at 4μrad. The measured points remain at or below the scale-plus-detector curve (for example 0.473 px against 0.530 px at 2μrad), the growing gap reflecting the additional information supplied by the bounded mirror-angle envelope. Over this range the correction error is controlled by the common-path reference and not by the network, so lowering it requires a better-compensated common path rather than a more accurate regressor.

### 4.2. Assumptions and Limitations

The floor and decomposition of Section 4.1 rely on the three assumptions of Section 2.4. We now quantify how each assumption’s violation degrades the estimate, so that the operating envelope is explicit rather than qualitative.

#### 4.2.1. Assumption 1 (Small-Angle Linearity)

Lemma 2 already shows that at the simulated envelope $\theta_{\max}=80\;\mu \mathrm{rad}$ the linearization error is $\leq \;9\times 10^{-8}\;\mathrm{px}$, negligible. The same bound predicts that the error grows only to $\approx \;2.2\times 10^{-5}\;\mathrm{px}$ at $\theta_{\max}=0.5\;\mathrm{mrad}$ and reaches the centroid displacement scale of 0.1px only near $\theta_{\max}\approx 8.3\;\mathrm{mrad}$. The method is therefore safe for any disturbance whose individual mirror angles stay below roughly 1mrad, which covers the μrad-to-sub-mrad regime of spacecraft micro-vibration; beyond that, the sensitivity $\Delta_{i}$ becomes angle dependent and the geometrically exact (nonlinear) model left for future work is required.

#### 4.2.2. Assumption 2 (Fixed Sensitivities)

If the calibrated sensitivities are in error by a fraction ε, i.e., $\Delta_{i}^{\mathrm{cal}}=\Delta_{i}^{\mathrm{true}}\left(1+\varepsilon \right)$, the recovery rule uses the wrong scale and injects $e_{\Delta }\approx -\varepsilon \sum_{i}\theta_{i}^{\prime }/\Delta_{i}^{\mathrm{true}}$, bounded by $|e_{\Delta }|\leq 3\;\theta_{\max}\;\varepsilon /\Delta_{\min}$. At the envelope used throughout this study ($\theta_{\max}=80\;\mu \mathrm{rad}$, Section 5) and ε=5% this worst-case bound evaluates to 0.52 px, not negligible against the 0.13 px correction error—but it assumes that all three non-shared mirrors sit simultaneously at the envelope with aligned signs. The exposure of the estimator evaluated here is narrower, because the network emits $q_{78}$ directly in pixel units, so that only the mirror-6 conversion and the metrology gain are exposed (Section 5.7); at the same ε=5% the RMS error realized on the simulated data is 0.097 px and the total RMSE grows from 0.130 to 0.162 px. A miscalibration of a few percent is therefore tolerable in practice—the error stays within 0.02 px for |ε|≲3%, and at ε=5% it adds only 0.03 px to the total—so periodic off-line recalibration is practical rather than urgent (Section 5.7). The sensitivities are established off-line by opto-mechanical calibration or ray tracing, exactly as for any FEA step, so recalibration after thermal drift or a structural reconfiguration is feasible and cheap relative to a full on-board re-solve.

#### 4.2.3. Assumption 3 (Matched Shared Path)

This is the most fragile. The shared telescope is designed with *equal* effective focal length in the two channels, so the 27.5% difference between the complete channels is confined to their post-split dedicated lens groups and supplies the non-shared-mirror differential sensitivity; it therefore injects *no* shared-path residual even when the shared mirrors vibrate. The genuine risk is an *alignment/calibration imbalance* β between the two channels’ shared-path magnifications: if the shared effective focal length differs by a fraction β, the cancellation in (1) is incomplete and a residual equal to β times the shared-element shift remains. The measurements of Section 5 show that this term, not the regression accuracy, is what limits the end-to-end result: the common-path residual enters the shift with $q_{\mathrm{sh}}=\beta \;C_{\mathrm{sh}}\mu$, whose RMS is $\beta \;C_{\mathrm{sh}}\;\mathrm{std}\left(\mu \right)=\beta \times 12.2\;\mathrm{px}$ for the simulated envelope, two orders of magnitude larger than the network error. The shared-path magnification match must therefore be verified at the sub-percent level during alignment, and any measured imbalance should be folded back into the calibrated $\Delta_{i}$ rather than treated as negligible.

For a quantitative sensitivity study, let the RMS common-mode image motion be $\sigma_{\mathrm{sh}}=C_{\mathrm{sh}}\mathrm{std}\left(\mu \right)=12.2$ px, and let the nominal proposed estimator error be $\sigma_{0}=0.130$ px, the measured correction RMSE. If the imbalance residual is uncorrelated with the nominal estimator error, then

(29) $$\mathrm{RMSE}\left(\beta \right)=\sqrt{\sigma_{0}^{2}+{\left(\beta \sigma_{\mathrm{sh}}\right)}^{2}}.$$

**Figure 3:**
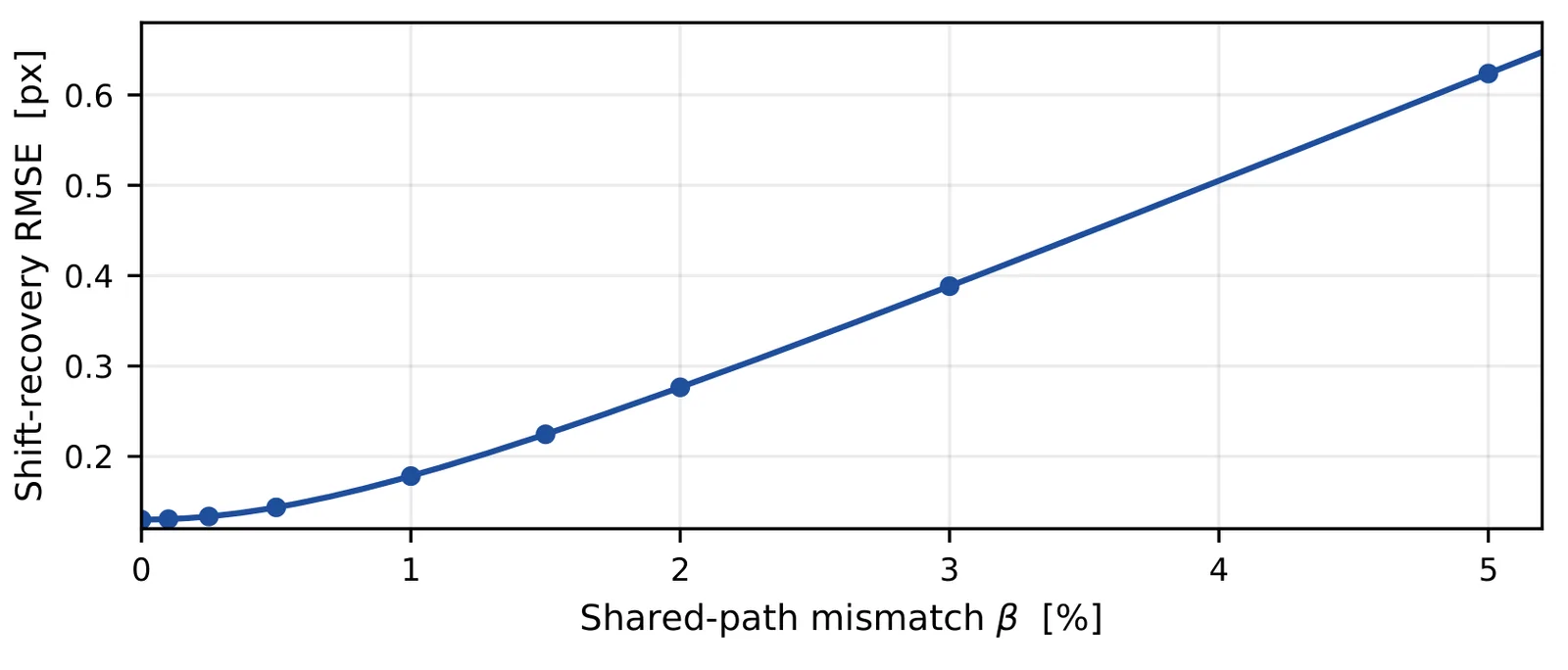
Analytical shared-path mismatch sensitivity under $\sigma_{\mathrm{sh}}=12.2$ px and $\sigma_{0}=0.130$ px.

Table 4 and Figure 3 evaluate the range β∈[0.1%,5%]. At β=0.5% the shared-path residual is 0.061 px and the total RMSE rises from 0.130 to 0.144 px; at β=1% it rises to 0.178 px, at 2% to 0.276 px, and at 5% to 0.623 px. The last value is the one used in the label definition of Section 5.6, where the independently measured total-correction error of 0.623 px confirms this analytical prediction. The sensitivity study therefore quantifies the penalty under the stated envelope and should not be interpreted as measured alignment performance; it also shows that the common-path match is a tighter requirement than any other element of the budget, since the mismatch dominates the error from β≈1% onwards.

Two practical limits lie outside the model. First, the centroid tracker can lose lock under low contrast or detector saturation, after which $\hat{P_{\mathrm{meas}}}$ is unavailable and the estimate must be flagged invalid until re-acquisition. Section 3.1 gives a quality-gating and bounded-hold policy (Algorithm 1); no temporal predictor is used. The linear baseline in Section 5.9 tests only flux/saturation gating and bounded holding under specified simulated faults; it does not validate the original neural pipeline. Second, the numerical results of Section 5 are obtained on a *simulated* dual-channel dataset. No independent ray-tracing validation, physical optical bench, hardware-in-the-loop test, cycle-accurate embedded hardware emulation, edge accelerator profiling, or on-orbit measurement is reported. Agreement with an analytical bound under the same model does not validate the physical optics or flight performance. The original neural network sensor noise analysis does not establish robustness to thermal optical distortion, spatially varying stray light, or sensor response non-uniformity. Section 5.9 adds defocus proxy, background gradient, and gain/offset simulations for a separately defined linear baseline. Instrument-specific thermal wavefront, scattering, and detector characterization remain outside these simplified simulations. The reported compute times are budget estimates; sensor exposure and readout, synchronization, transfer, centroid tracking, quality gating, and post-processing must be measured before an end-to-end frame rate can be claimed.

## 5. Numerical Experiments

All results in this section are obtained on a *simulated* dual-channel dataset generated by the measurement forward model of Section 3.3; no ray trace of the stated prescription, optical bench, hardware-in-the-loop test, or on-orbit measurement is used. The auxiliary mirror-6 term is supplied analytically under the stated noise model and is not synthesized as an additional image. Because independent mirror-6 motion is not identifiable from the measurement image (Section 2.3), the experiment evaluates the *observable* correction $q_{78}$ together with that auxiliary term. Section 5.9 reports a separately defined linear baseline and does not validate the estimator studied here.

### 5.1. Dataset and Protocol

The renderer follows Section 3.3 and each sample is one 64×64 measurement patch. Consecutive frames are read out at fixed detector coordinates, so the patch contains the tracked translation instead of removing it by recentering. Mirror angles are drawn independently and uniformly from [−80,80]μrad, and the front-end common-path compensation leaves a residual $\delta \mu ∼N(0,\sigma_{\delta \mu }^{2})$ with $\sigma_{\delta \mu }=0.5\;\mu$rad. The common-path term $C_{\mathrm{sh}}\mu$ itself is shared by the tracked displacement and by the ground-truth imaging shift and cancels in the error statistics; what the renderer has to reproduce is the residual that survives compensation, and the sensitivity of the result to the matched-path assumption is quantified in Section 4.2 and revisited at the end of Section 5.6.

On the simulated dat, the ground-truth correction $q_{78}=\theta_{7}^{\prime }/\Delta_{7}+\theta_{8}^{\prime }/\Delta_{8}$ has a standard deviation of 1.87 px and the auxiliary mirror-6 term has an RMS of 2.03 px, so the correction and the auxiliary term are of the same order and neither is negligible. Training uses 20,000 patches per epoch drawn on-line, a fixed validation set of 5000 patches, and a fixed test set of 10,000 patches; it converges in about 4 min on an RTX 5060 Laptop GPU with the training and validation curves tracking each other.

Two evaluation protocols are used, and the two are never mixed within one comparison. The accuracy, calibration, and complexity results are computed on the fixed 10,000-patch test set, with 95% percentile bootstrap intervals from 10,000 resamples of *samples*. The time-domain, frequency-domain, noise robustness, and in-envelope temporal waveform studies use 20 independent 400-frame (0.2 s) band-limited trajectories per condition, which are closer to an operational record, and resample whole *trajectories*; the intervals are correspondingly wider. Those studies use the full-precision network, and quantization and pruning are ablated separately in Section 5.8.

### 5.2. Baselines and Metrics

Four estimators are compared. (i) *Centroid-only* reports $\hat{P_{\mathrm{img}}}=\hat{P_{\mathrm{meas}}}$ and therefore ignores the non-shared mirrors entirely; its tracker is a threshold-weighted centroid computed after fitting and removing a background plane. (ii) An *FEA-style surrogate* passes the mirror angles through an off-line structural model carrying a 12% scale error, a 1μrad bias, and 1.2μrad independent noise (a=0.88, b=1μrad, σ=1.2μrad); it is an illustrative pre-update mismatch scenario rather than a calibrated finite element characterization, and it is deliberately driven by the true mirror angles so that it isolates the mismatch contribution instead of compounding it with the state-estimation error that a real structural model would also incur. Published sim-to-test and finite element update discrepancies motivate assessing such a scenario [10,46,47], but the selected parameters require validation for any particular instrument. (iii) The proposed network at full precision and (iv) the proposed network after INT8 quantization (Section 3.2). Accuracy of the observable correction $q_{78}$ is reported as RMSE and MAE in pixels; recovered imaging channel shift is reported separately as RMSE. The pruning ratio is treated as a design knob and ablated in Section 5.8.

### 5.3. Accuracy at the Nominal Operating Point

Table 5 reports the accuracy on the fixed test set at the nominal operating point. Centroid-only tracking is in error by 0.396 px, because the non-shared-mirror contribution is not removed from the measurement channel displacement. The FEA-style surrogate reduces the error to 0.236 px but retains the mismatch residue. The proposed network reaches 0.1298 px [0.1280, 0.1317], a 3.0× reduction against the tracking baseline and a 1.8× reduction against the surrogate; INT8 quantization raises the error to 0.1334 px [0.1315, 0.1353], a penalty of 0.004 px.

An oracle that receives the true measurement channel displacement, and therefore carries no tracking error, attains 0.1309 px [0.1290, 0.1327]. The three values 0.1298, 0.1309, and the structural scale $C_{\mathrm{sh}}\sigma_{\delta \mu }=0.1318$ px agree to within the resampling interval, so the trained regressor already operates at the structural scale of the correction derived in Section 2.3: the residual error is set by the common-path compensation residual rather than by the capacity of the network.

Substituting the network into the recovery rule (1) gives an end-to-end RMSE of 0.3976 px for the imaging channel shift. The independent-terms budget of Lemma 1 predicts $\sqrt{0.3779^{2}+0.002^{2}+0.1298^{2}}=0.400$ px from the three measured components—the tracking error 0.378 px, the auxiliary metrology term 0.002 px at $\sigma_{6}=0.05\;\mu$rad, and the correction error 0.130 px—so the budget reproduces the measured end-to-end error to within 1%. The tracking term exceeds the correction term by a factor of 2.9, so the end-to-end accuracy is limited by the displacement tracking stage and not by the regressor: with a perfect displacement measurement the same budget would give 0.131 px.

Because that budget is a model, its independence assumption has to be checked rather than presumed. For the proposed estimator, the two dominant terms are effectively uncorrelated and the agreement above is within 1%. The check is informative precisely where the assumption fails. The FEA-style surrogate is driven by the true mirror angles through a mismatched model, so its mirror-6 term is also perturbed, with an RMS of 0.253 px; inserting the three measured terms (0.378,0.253,0.236) px into the same quadrature gives 0.512 px against a measured end-to-end value of 0.375 px. The centroid-only estimator supplies neither the auxiliary term nor the correction, so its three terms are (0.378,2.03,1.87) px and the same quadrature gives 2.78 px against 2.62 px. In both cases, the quadrature overestimates the measured error because the omitted terms are negatively correlated with the tracking error on this dataset; the pairing of residual components with independent variances is therefore a property of the proposed estimator and not of the baselines. The method comparison is therefore reported on the correction error $q_{78}$, which is the quantity each estimator is asked to predict, and not on the end-to-end shift, whose ranking is not determined by estimator accuracy alone. This is the caveat that Section 4.1 attaches to Lemma 1, now with measured magnitudes.

**Figure 4:**
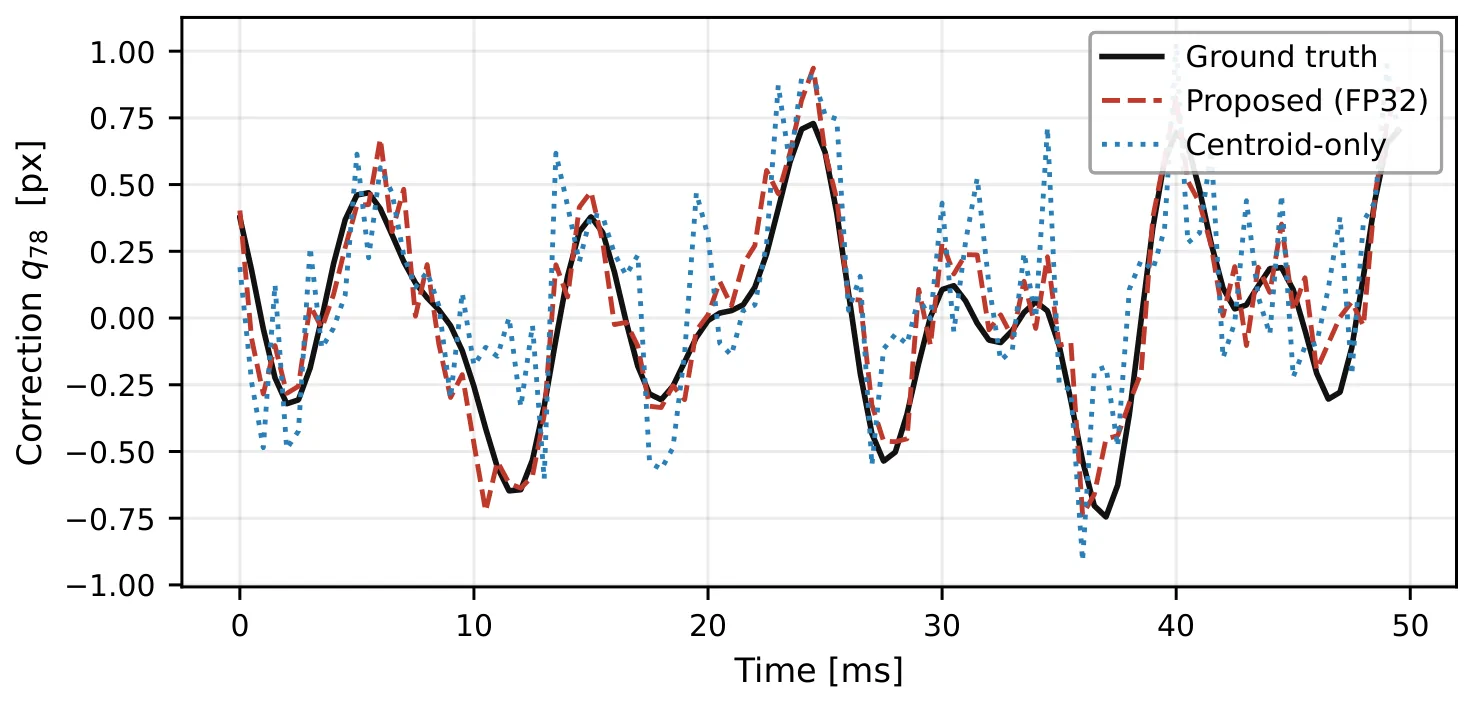
Observable correction $q_{78}$ over a 50 ms window of a held-out trajectory: ground truth, the proposed estimate, and the centroid-only estimate, which is offset by the unremoved non-shared-mirror contribution.

**Figure 5:**
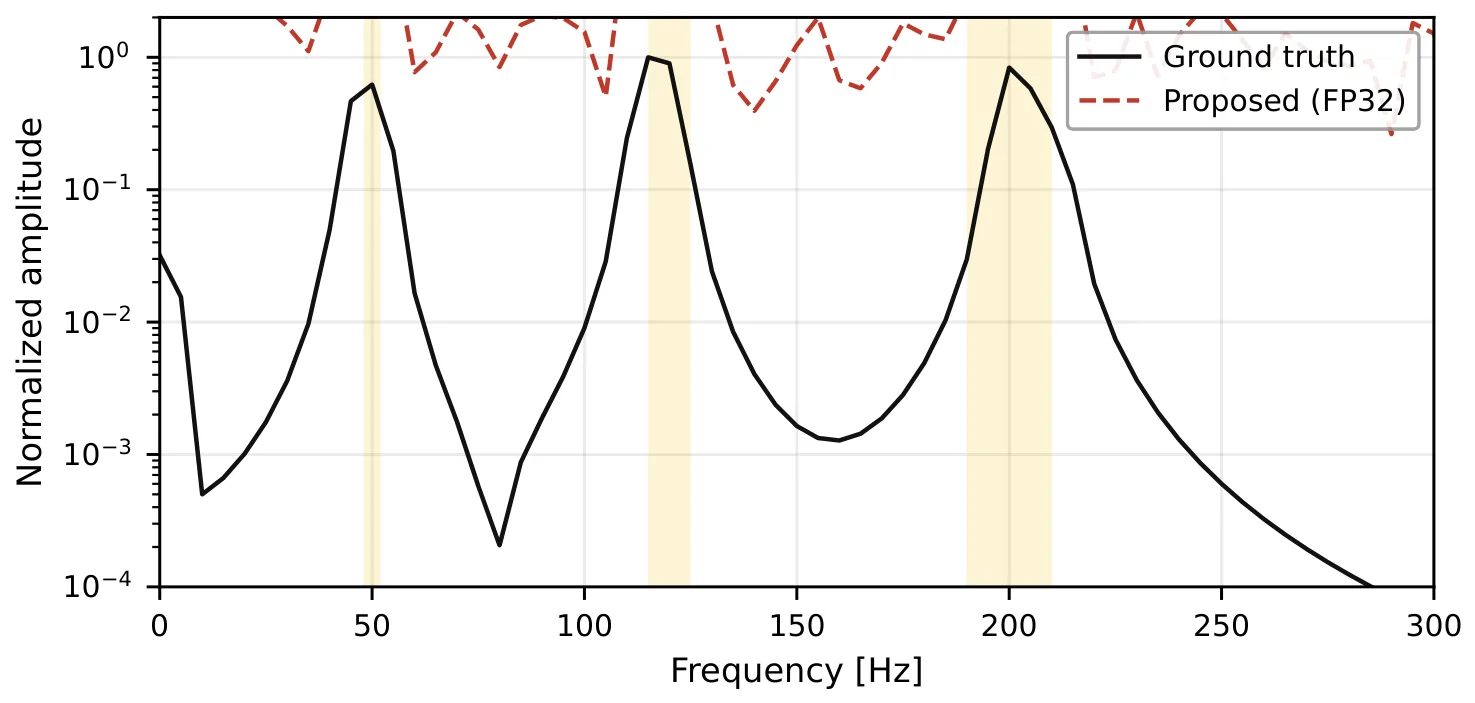
Normalized spectrum of the true and estimated correction; the shaded bands mark the three training bands in which lines are injected.

**Figure 6:**
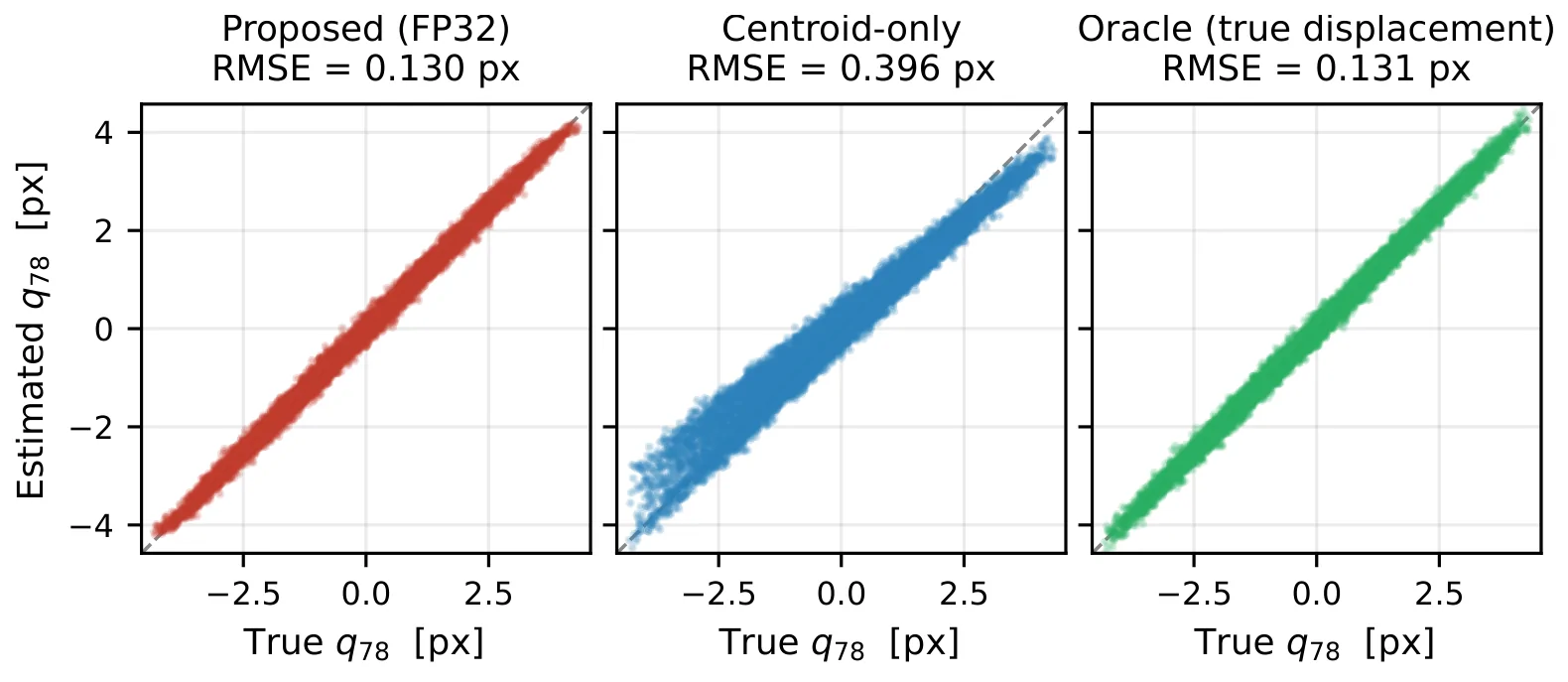
Predicted versus true observable correction $q_{78}$ on the held-out test set: the proposed network, the centroid-only tracker, and the oracle that uses the true measurement channel displacement.

Figure 4 shows a 50 ms window of a held-out trajectory: the proposed estimate follows the ground-truth correction, whereas the centroid-only trace carries the unremoved mirror contribution. Figure 5 shows the corresponding spectrum. Lines are injected only in the three training bands (48–52, 115–125, and 190–210 Hz), and the estimated spectrum reproduces them at the same amplitudes as the truth; away from the lines, the estimate stays close to the true spectrum down to the noise floor of the record. Figure 6 shows the predicted versus true observable correction on the held-out test set for the proposed network, the centroid-only tracker, and the oracle, with the RMSE of each estimator annotated in the corresponding panel.

### 5.4. Robustness to Sensor Noise

**Figure 7:**
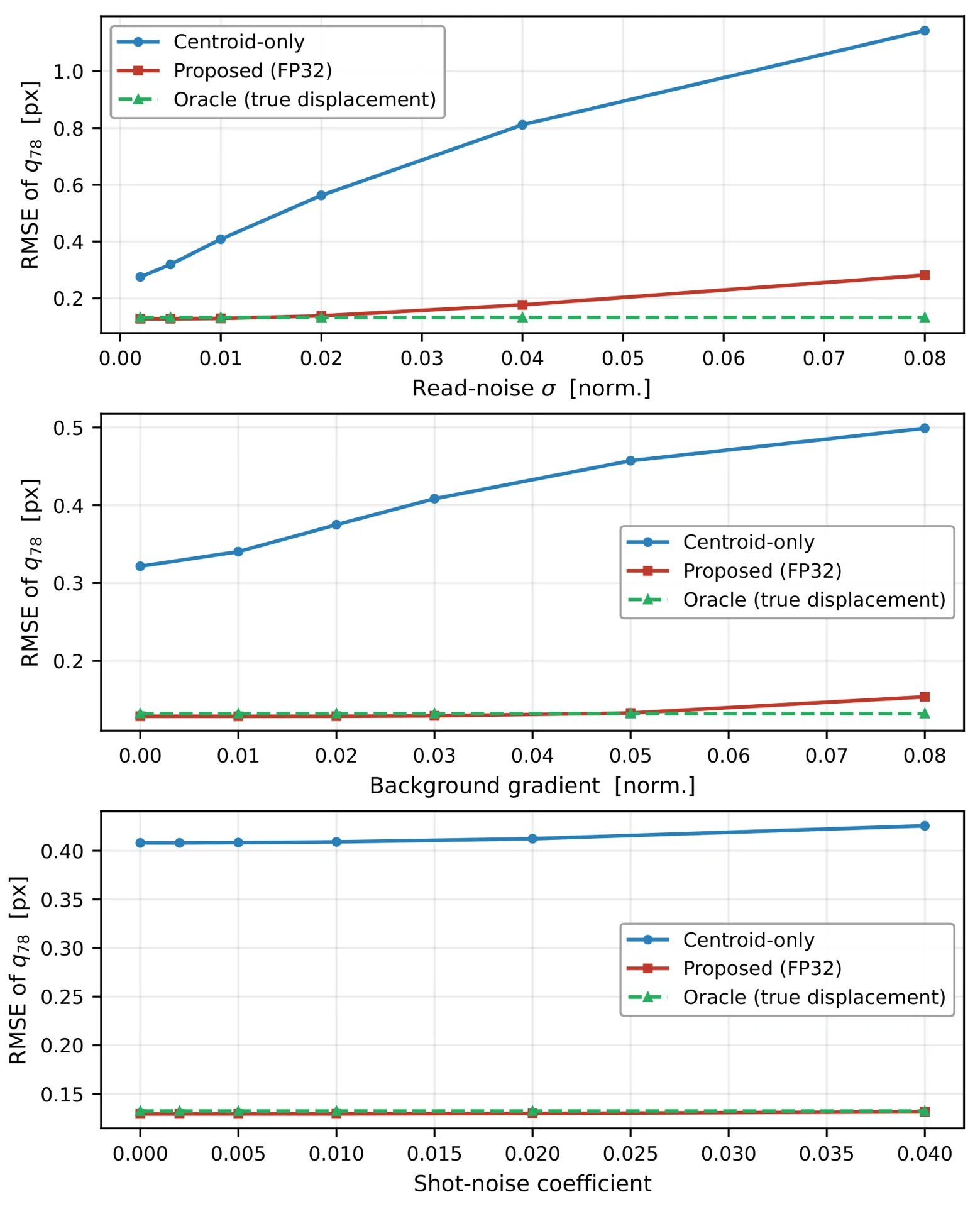
RMSE of the observable correction versus read noise, background gradient amplitude (middle), and shot-noise coefficient, each swept with the other settings held at the nominal point. The dashed curve is the oracle that uses the true measurement channel displacement.

Figure 7 sweeps the three detector settings of the renderer one at a time around the nominal point. Against read noise (σ from 0.002 to 0.08 of full scale) the centroid error grows from 0.28 to 1.14 px, whereas the network grows only from 0.128 to 0.282 px and matches the oracle value of 0.132 px up to the nominal σ=0.01; at σ=0.02 it is 4.1× more accurate than the tracker. Against a background gradient (0 to 0.08 of full scale) the centroid error grows from 0.32 to 0.50 px and the network from 0.129 to 0.154 px, a factor of 3.2 at the nominal gradient of 0.03. The shot-noise coefficient (0 to 0.04) changes neither estimator materially at these signal levels (0.408 to 0.426 px for the centroid, 0.129 to 0.132 px for the network). The insensitivity to background gradient and read noise is expected: the network uses the whole patch, whereas a centroid is a first-moment statistic that is directly biased by the background and directly perturbed by per-pixel noise. These are simulation settings rather than detector specifications, and the sweep does not cover stray light, thermal wavefront error, or response non-linearity beyond the modeled PRNU and DSNU.

### 5.5. Unseen Temporal Waveform Generalization Within the Training Envelope

Table 6 tests three temporal waveform families that were not used in the trajectory studies during training, without retraining: lower and higher frequency bands at the same amplitudes, and a mixed waveform composed of a 40–70 Hz chirp, a 280 Hz line, and sparse impulses. The network error is statistically indistinguishable across the conditions (0.130–0.132 px, against 0.132 px in distribution), and the centroid baseline is equally unchanged (0.28–0.29 px).

This behavior follows from the regression target rather than from a general robustness property. The correction depends on mirrors 7 and 8 only through the scalar $q_{78}$, and the amplitudes were sampled uniformly over the operative envelope, so changing the frequency content or waveform at fixed amplitude does not create a strict input distribution shift. The test is thus an in-envelope temporal waveform generalization check; it does not establish generalization to amplitudes, detector conditions, or optical configurations outside the trained envelope, and it does not replace the ray-trace and bench validation noted in Section 4.2.

### 5.6. Ablation and On-Board Complexity

**Figure 8:**
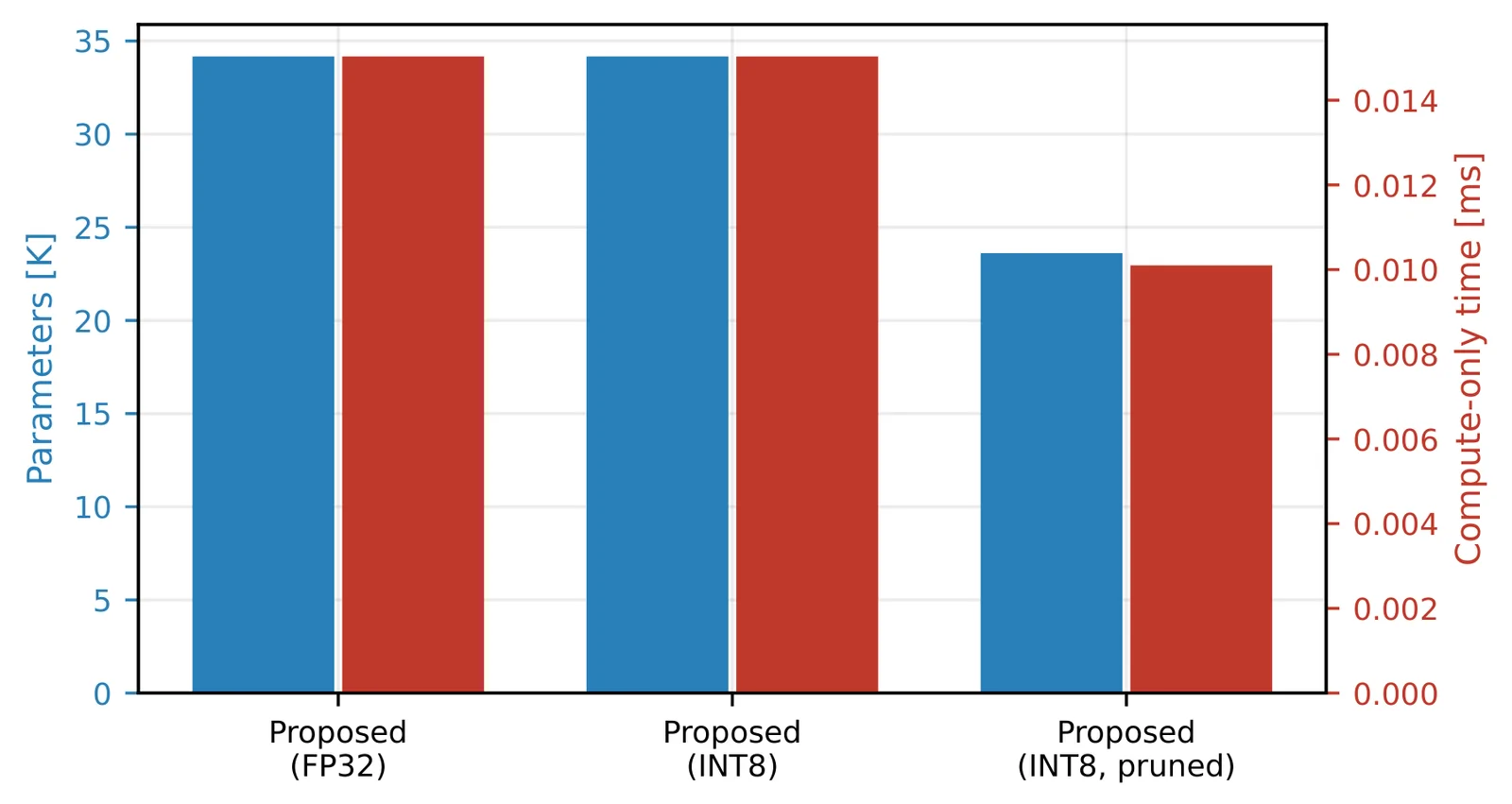
Parameter count (**left axis**) and projected compute-only time (**right axis**) for three network configurations. The plotted times are not hardware measurements.

Throughout this paper one multiply–accumulate of a weight–activation pair is counted as one MAC, and the projected time assumes that one MAC costs two operations at the stated INT8 throughput. Under that convention, the full-precision network performs $9.02\times 10^{5}$ MACs per frame, i.e., $9.0\times 10^{-4}$ G, and the count is dominated by the $16^{2}$ stage (51.8% of the total) rather than by the $64^{2}$ input stage (4.7%). Table 7 and Figure 8 summarize the sizes and the projected compute-only times. Retaining 80% of the channels per stage lowers the count to $6.06\times 10^{-4}$ G, and the corresponding compute-only times are 0.0150 ms and 0.0101 ms per frame at 120 GOPS, i.e., 3.0% and 2.0% of the 0.5 ms frame interval.

These are arithmetic budget figures, not measured latencies: memory traffic, scheduling, synchronization, and the remaining pipeline stages are excluded, and a network of this size on an embedded target is frequently memory-bound rather than MAC-bound. A budget of this magnitude does not by itself establish that the 2000 Hz frame rate can be closed; what it does establish is that the regressor is no longer the dominant term of the on-board budget, in contrast to a full finite element re-solve.

**Figure 9:**
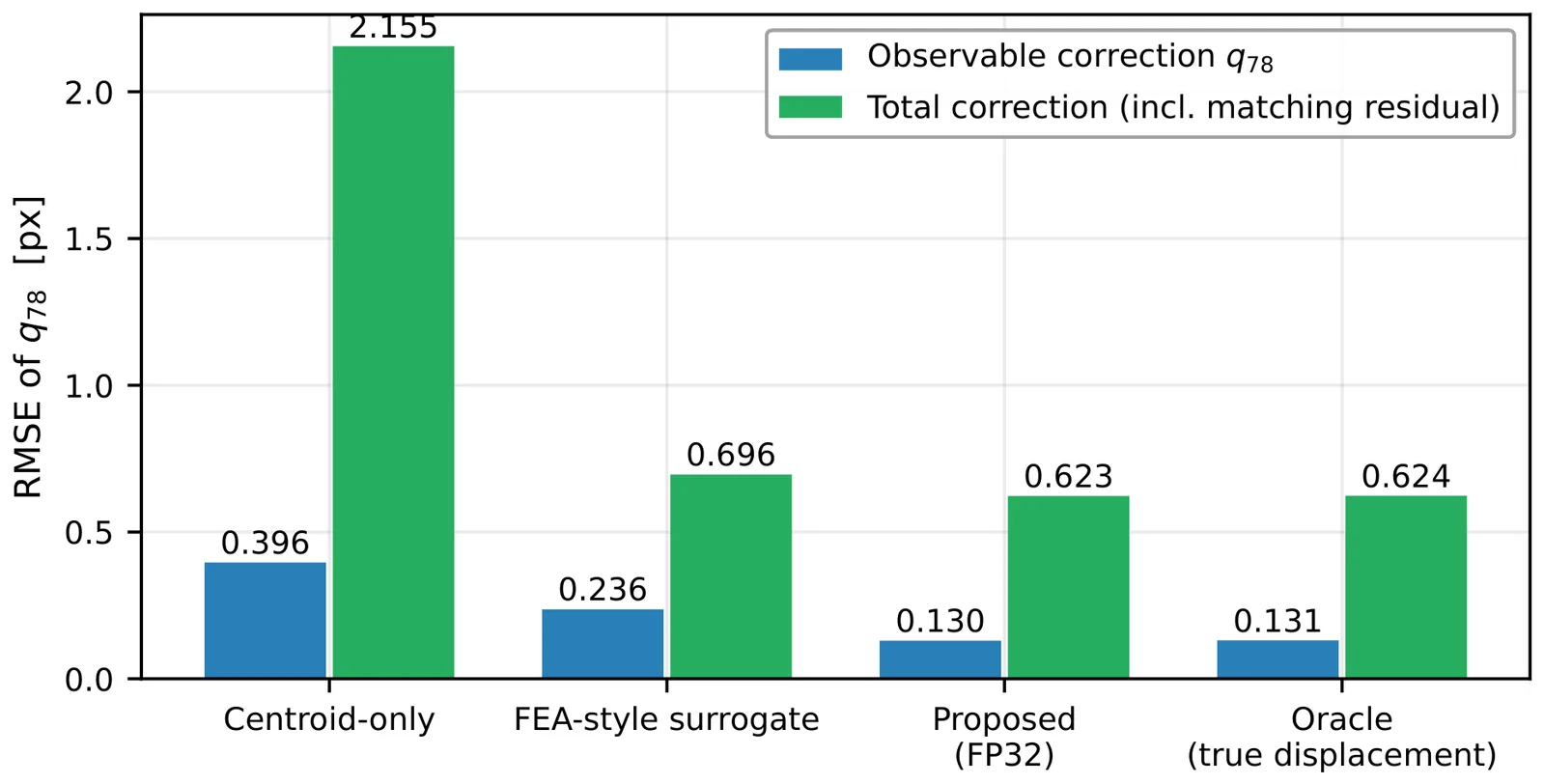
RMSE at the nominal operating point of the observable correction $q_{78}$ and of the total correction, the latter including the channel-matching residual of Assumption 3.

Figure 9 also reports RMSE of the total correction, which additionally contains the channel-matching residual $\beta C_{\mathrm{sh}}\mu$ of Assumption 3. That residual is not present in the rendered image—the patch contains only the compensated residual δμ—so it is reported separately rather than folded into the end-to-end RMSE. At the β=5% value used in the label definition its RMS is 0.61 px, and the measured total-correction RMSE of the proposed estimator is 0.623 px, within 2% of the analytical prediction of Table 4. The network barely improves on the surrogate (0.696 px) in this column because this error component is not reducible by any estimator that sees the measurement image alone. This is the quantitative statement of the shared-path requirement discussed in Section 4.2: at the assumed matching level the end-to-end accuracy would be set by the channel match, not by the regressor.

### 5.7. Sensitivity to Calibration Errors

**Figure 10:**
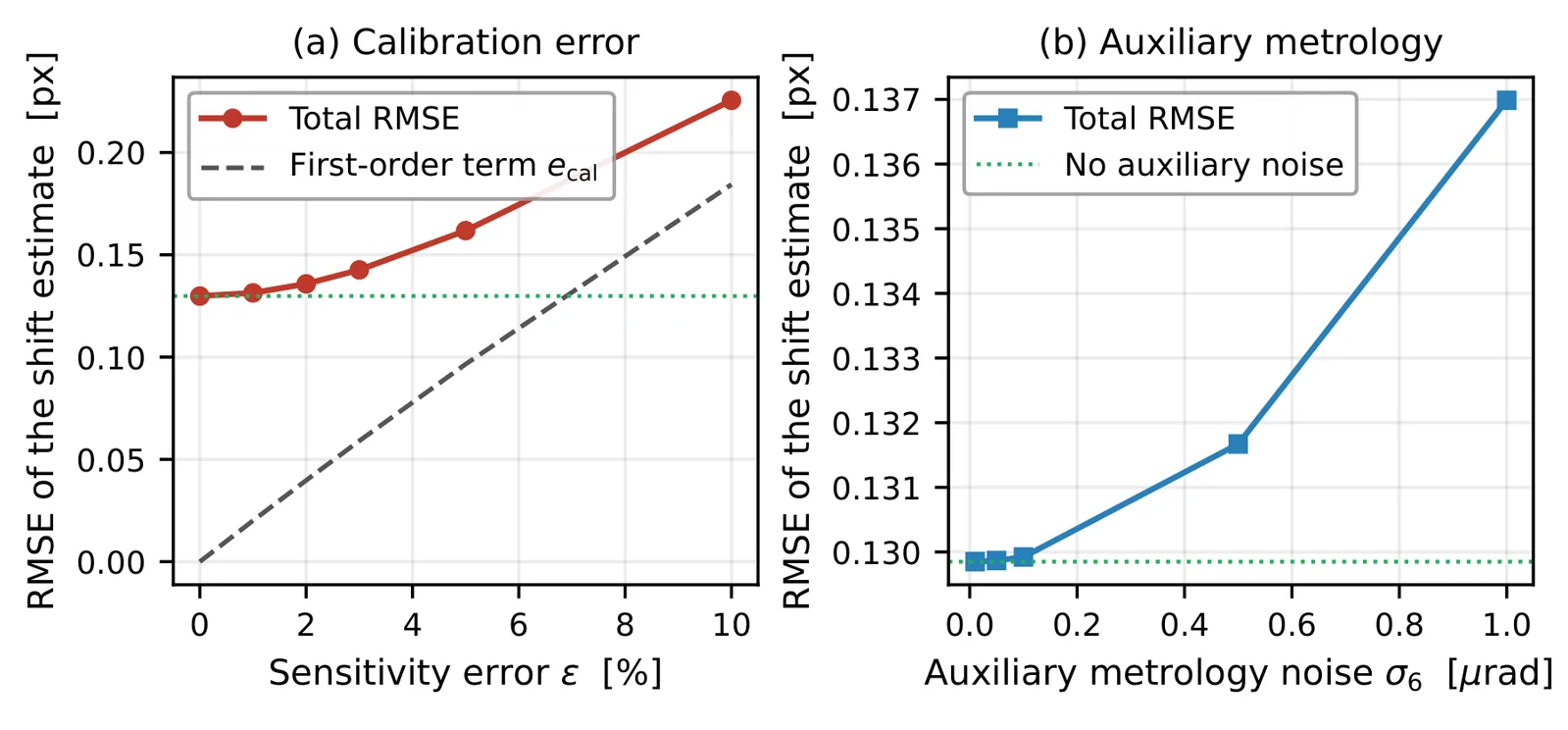
(**a**) RMSE of the shift estimate versus relative sensitivity error ε; the dashed curve is the first-order term (30) and the dotted line is the perfectly calibrated value. (**b**) Same RMSE versus auxiliary mirror-6 metrology noise $\sigma_{6}$.

The auxiliary mirror-6 term is ${\hat{q}}_{6}=a_{6}/\left(g_{6}\Delta_{6}\right)$, so a relative error ε in the calibrated sensitivity (used value $\Delta_{6}^{\mathrm{used}}=\Delta_{6}\left(1+\varepsilon \right)$) injects

(30) $$e_{\mathrm{cal}}=-\frac{\varepsilon }{1+\varepsilon }\;\frac{\hat{\theta_{6}^{\prime }}}{\Delta_{6}},$$

whose RMS is |ε|/(1+ε) times the RMS of the mirror-6 term, measured on the simulated data as $\sigma_{\mathrm{corr}}=2.03$ px. Because the network is trained to emit the correction $q_{78}$ directly in pixel units, a mis-calibration of $\Delta_{7}$ or $\Delta_{8}$ does not propagate at inference; the exposed calibrations are the mirror-6 conversion above and the metrology gain $g_{6}$. Figure 10a shows the resulting sensitivity: the total RMSE rises from 0.130 px at perfect calibration to 0.132, 0.136, and 0.143 px for ε=1%, 2%, and 3%, and to 0.162 and 0.226 px for ε=5% and 10%. Off-line recalibration of $\Delta_{6}$ is therefore practical rather than urgent: the error stays within 0.02 px as long as |ε|≲3%.

Figure 10b sweeps the auxiliary metrology noise. Between $\sigma_{6}=0.01$ and 1.00μrad the mirror-6 contribution grows from 0.0004 to 0.044 px, and the total RMSE moves only from 0.1299 to 0.1370 px, because the auxiliary term enters the budget in quadrature with the 0.13 px correction error. Auxiliary metrology is therefore not a limiting term as long as $\sigma_{6}≲1\;\mu$rad. The two sweeps of this subsection are simulation settings, not instrument specifications.

### 5.8. Bit-Width and Pruning Ablation

**Figure 11:**
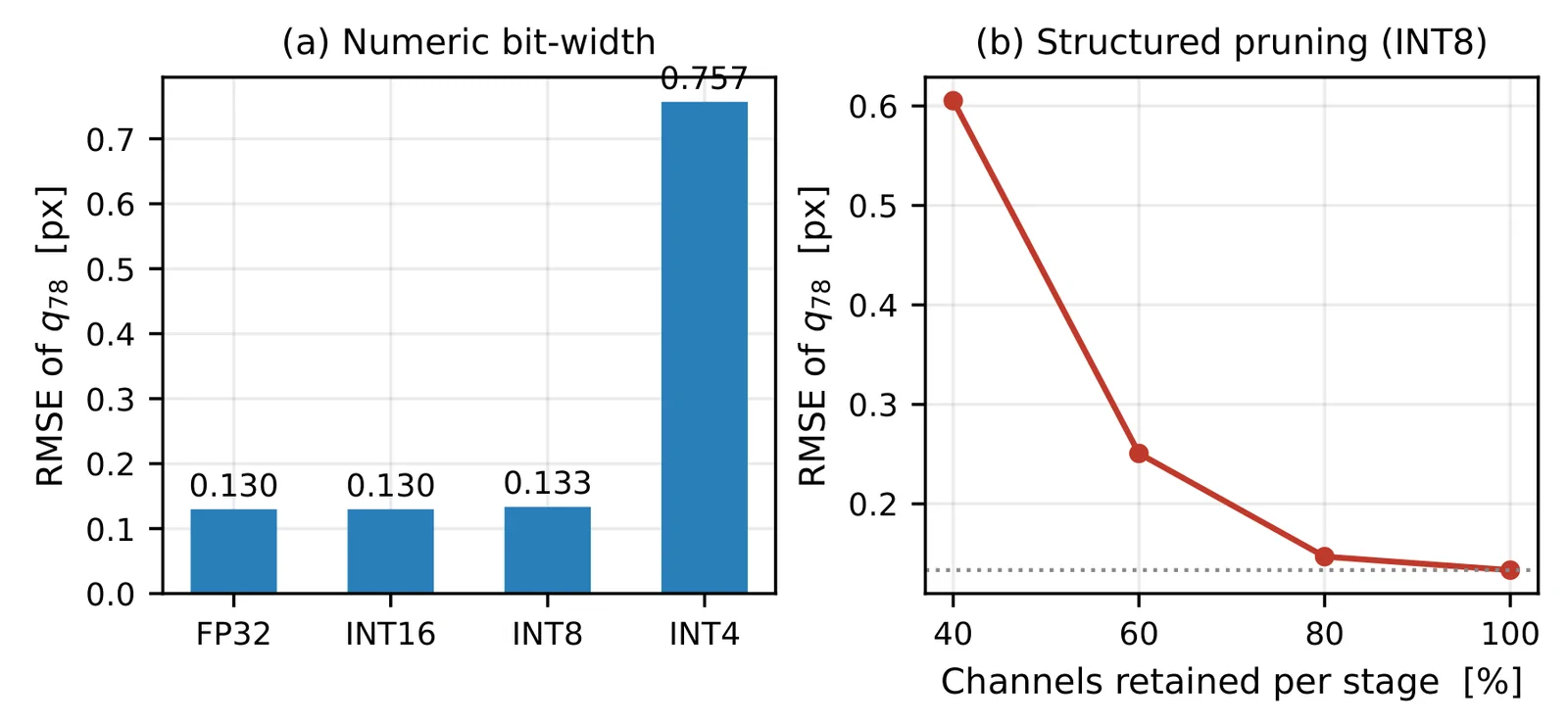
(**a**) Correction RMSE versus numeric bit-width. (**b**) Correction RMSE versus the fraction of output channels retained per stage at fixed INT8.

Two implementation choices determine the deployed size: the numeric bit-width and the fraction of channels retained. Figure 11a varies the weight/activation precision. FP32 and FP16 are indistinguishable (0.1298 px both), because the regression target is smooth and low-dimensional. INT8 costs 0.004 px (0.1334 px, +3%), whereas INT4 collapses to 0.757 px. INT8 is therefore the last precision before accuracy degrades faster than the stored footprint improves.

Figure 11b varies the fraction of channels retained per stage at fixed INT8, and the resulting accuracy–compute curve is not uniform. Removing the first 20% of the channels costs 0.014 px and saves 33% of the MACs (0.1334→0.1470 px and $9.02\;\times \;10^{-4}\;\to \;$ $6.06\;\times \;10^{-4}$ G); the next 20% costs 0.104 px (→0.2507 px, $3.67\;\times \;10^{-4}$ G); and retaining only 40% costs a further 0.355 px and is not usable at the required accuracy (0.605 px, $1.90\;\times \;10^{-4}$ G). The knee of the trade-off is therefore at 80% retained, the smallest configuration that we would deploy: 23,614 parameters, $6.06\;\times \;10^{-4}$ G MAC, a 0.024 MB stored footprint, and a projected compute-only time of 0.0101 ms. The accuracy tables use the unpruned INT8 network; adopting the pruned variant costs 0.014 px of correction RMSE.

### 5.9. Additional Validation of an Explicitly Observed Linear Baseline

The following newly executed simulations address non-ideal image formation, tracking faults, error correlation, and sensitivity to assumed model mismatch. They evaluate a linear estimator with explicitly supplied auxiliary observations, not the original neural network. The original image-only results above do not establish the performance of this extension. Conversely, the results below do not validate the original three-angle neural inference or a constructed instrument.

#### 5.9.1. Observation Model and Parameter Provenance

Three separate auxiliary metrology spots measure the non-shared mirror angles. For $\theta_{i}^{\prime }$ expressed numerically in μrad, the noiseless spot displacements and main-channel shifts, in pixels, are

(31) $$u_{i}=0.1\theta_{i}^{\prime }\;\left(i=6,7,8\right),\;P_{\mathrm{meas}}=s-0.1\theta_{7}^{\prime }-0.1\theta_{8}^{\prime },\;P_{\mathrm{img}}=s+0.1\theta_{6}^{\prime }.$$

Here *s* is shared-path motion. The estimator adds the measured centroid displacements: ${\hat{P}}_{\mathrm{img}}={\hat{P}}_{\mathrm{meas}}+{\hat{u}}_{6}+{\hat{u}}_{7}+{\hat{u}}_{8}$. The auxiliary paths make the otherwise hidden angular states observable by construction. This is a sufficient reference configuration, not a claim of the minimum sensor count. Its signed coefficients are assumed nominal values consistent with the paper’s synthetic correction scale. An unfolded free-space metrology path with a 0.75 m angular lever arm and 15 μm pixel pitch gives 2L/p=0.1 px/μrad. Neither that lever arm nor the auxiliary metrology paths are taken from the engineering design report. The metrology geometry of this subsection is therefore distinct from the geometry-derived sensitivities (6) used in Section 5, and the two sets of coefficients are not interchangeable; this subsection is retained as an independently specified benchmark with its own stated lever arm.

The supplied dual-channel optical design report provides a 4331 mm focal length, 1300 mm entrance pupil, and 15 μm pixel-based image quality evaluation. It also reports approximately ±0.004 mm focus adjustment for uniform ±3 K changes, and 0.044 mm/K focus sensitivity for one short-wave relay lens. These values anchor the additional scale and defocus ranges only. That report describes short-/mid-wave infrared channels, not the paper’s visible measurement branch; its prescription and PSF are not reconstructed here. All other settings in Table 8 are explicit simulation assumptions.

Random seed defines 20 independent phase/amplitude/frequency draws; the same trajectories are used across conditions. The estimator has no training stage and no parameter selection on these records. Reference coordinates are assumed known. Fixed gain and offset maps are drawn per trajectory and sensor; photon noise is Poisson and read noise Gaussian. A common time-dependent background gradient acts across the four sensors. Uniform temperature is quasi-static within each trajectory and spans −3 to +3 K across records; the single-lens case spans −1 to +1 K. With F#=4331/1300, a defocus proxy uses $\sigma^{2}=\sigma_{0}^{2}+{\left[\delta z/\left(4F\#p\right)\right]}^{2}$, matching the per-axis variance of a geometrical defocus disk. It approximates symmetric broadening, not the full thermal wavefront or field distortion. Instantaneous spots are sampled on the pixel grid and normalized to the signal budget. The exposure image and ground-truth displacement use the same three temporal sub-samples. Pixel-area integration and a wavelength-resolved PSF are not modeled.

#### 5.9.2. Image Non-Idealities and Fault Handling

Two centroid estimators subtract either a median border background or a fitted background plane. Both use a fixed 15×15 aperture and retain positive residual pixels above 9 electrons. The plane is fitted to border pixels with |x|≥9 or |y|≥9. A frame is rejected if any sensor has residual flux at most 1000 electrons or a maximum pixel value at least 1999 electrons. Thresholds are fixed simulation settings, not flight-qualified values. Each trajectory in the fault condition contains four eight-frame events in the main measurement sensor: a zeroed frame block, a bright single-pixel artifact, a saturated 3×3 patch, and target disappearance into noise. The last is a severe low-contrast limit rather than a full contrast sweep. The last valid output is held for at most three frames (1.5 ms), after which the output is invalid until the next accepted frame. All metrics count held outputs as available and retain their errors; unavailable outputs are excluded from conditional RMSE and explicitly counted in availability.

Table 9 reports means of trajectory RMSEs, with 95% percentile intervals from 10,000 whole-trajectory bootstrap resamples. These are uncertainty intervals for this new baseline only.

The uniform and local thermal proxy cases do not show a substantial penalty in this range; symmetric defocus primarily changes width rather than noiseless centroid position. This does not demonstrate immunity to thermal distortion. The gradient case is more demanding for constant-background subtraction, whereas the fitted plane reduces its bias. Non-uniformity and model/threshold effects remain after that subtraction.

**Figure 12:**
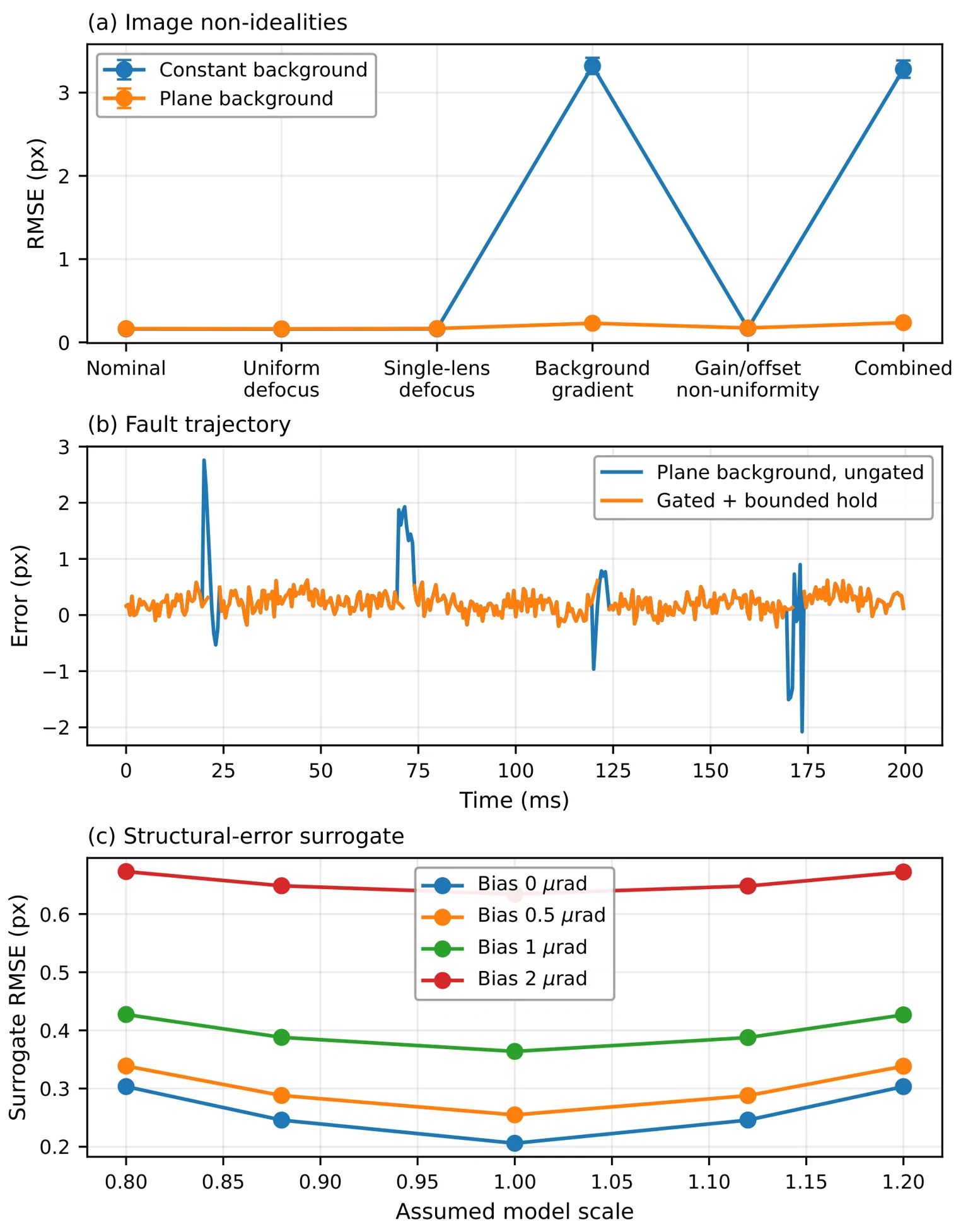
Executed additional studies. (**a**) Image condition RMSE and trajectory bootstrap intervals for two background estimators. (**b**) An example fault trajectory with the same plane background estimator before and after gating; gaps denote unavailable outputs. (**c**) Assumed structural error sensitivity at σ=1.2μrad. No panel evaluates the original neural network or actual FEA hardware.

With the same plane background estimator, the fault condition ungated RMSE is 0.371 px, and gated/held conditional RMSE is 0.238 px at 95.0% output availability (92% directly accepted frames). All injected faulty frames are rejected and no clean frames are rejected in these runs. This reflects the deliberately separable severe events and fixed thresholds, not a universal detection guarantee. Measurement updates resume on the first clean frame following each event (0.5 ms after the last faulty frame); processor latency is not included. Figure 12 shows the recovery behavior and the conditional nature of the improvement.

#### 5.9.3. Empirical Covariance Budget

The four errors are measured from the rendered-image centroid estimates, not generated as independent error terms. Let e contain the measurement displacement error and the three auxiliary displacement errors. For each full trajectory before fault gating, compute its mean μ and population covariance C. The exact empirical identity is

(32) $$\mathrm{MSE}\left({\hat{P}}_{\mathrm{img}}\right)=1^{T}C1+{\left(1^{T}\mu \right)}^{2}.$$

It agrees with direct residual MSE to numerical precision. Table 10 lists the resulting per-trajectory budgets. The simpler trC omits both cross-covariances and bias.

These results show why the independence approximation cannot be presumed even for this simple estimator. Correlation and bias vary with the image model; this test does not verify the covariance of the original neural network errors.

#### 5.9.4. Assumed Structural Model Mismatch Sweep

No finite element solve is introduced. Instead, the error surrogate ${\hat{\theta }}_{i}^{\prime }=a\theta_{i}^{\prime }+b+\epsilon_{i}$, with independent $\epsilon_{i}∼N\left(0,\sigma^{2}\right)$, tests the sensitivity of the paper’s assumed mismatch comparison. The sweep uses a∈{0.80,0.88,1.00,1.12,1.20}, b∈{0,0.5,1,2}μrad, and σ∈{0,0.5,1.2,2}μrad: 80 settings, each on 20 trajectories. The correction error is $0.1\sum_{i}\left[\left(a-1\right)\theta_{i}^{\prime }+b+\epsilon_{i}\right]$ pixels; measurement channel noise is excluded here to isolate model mismatch. At the earlier assumed setting (a,b,σ)=(0.88,1,1.2), the newly computed mean RMSE is 0.388 [0.383, 0.393] px, compared with 0.389 px from conditional analytical noise propagation. This is a new surrogate result on a different evaluation protocol, not a reproduction of the FEA-style row of Table 5: that row uses the geometry-derived sensitivities (6) together with the network’s protocol and target, so the two values differ in sensitivity scale, estimator, and target definition, and neither supersedes the other. An exact noise-free model gives zero mismatch error, whereas increasing bias or noise raises the error. Hence, the comparison depends on the selected mismatch; it cannot establish universal superiority over FEA.

#### 5.9.5. A Geometry-Derived Observability Example

As a separate paraxial example, consider two flat-mirror angular kicks with separation *d*, and two observation planes at distances $L_{1},L_{2}$ from the first mirror along the unfolded path. With shared motion known or separately removed, $z=B{\left(\theta_{7}^{\prime },\theta_{8}^{\prime }\right)}^{T}$ and

(33) $$B=\frac{2}{p}\left[\begin{array}{cc} L_{1} & L_{1}-d\\ L_{2} & L_{2}-d \end{array}\right],\;\det B=\frac{4d(L_{2}-L_{1})}{p^{2}}.$$

For assumed d=0.05 m, $L_{1}=0.10$ m, $L_{2}=0.20$ m and p=15μm, the matrix is $\left[\begin{array}{cc} 13333.3 & 6666.7\\ 26666.7 & 20000.0 \end{array}\right]$ in px/rad, with condition number 14.93 under equal independent spot noise and equal angular scaling. Coincident observation planes give a rank of one. Numerical inversion of 2000 independent angle pairs in [−13,13]μrad recovers the noiseless states to numerical precision. At 0.005 px independent spot noise, mirror-7/8 RMSEs become 1.22 and 1.72 μrad, illustrating noise amplification despite full rank. This is a geometry-derived example and not a ray trace of the report’s optical prescription. Unknown shared motion would require additional nuisance observations.

A separate 100,000-sample auxiliary noise check at each of 0.01, 0.05, 0.10, 0.50, and 1.00μrad confirms the expected 0.1σ pixel RMS contribution to within 0.15% in these draws. This supports the stated conditional noise budget, not the achievable precision of any sensor. Optical bench validation and hardware timing remain outside the evidence provided by these simulations.

## 6. Conclusions

This paper presented a simulation study of an optical measurement-assisted neural regression method for estimating micro-vibration image shift in a space camera. The proposed dual-channel architecture shares the front optics between an imaging channel and a high-frame-rate measurement channel, while a lightweight conventional neural regressor estimates the observable correction formed by the two measurement branch mirrors in the synthetic study. The separate branch optical layout cannot identify independent motion of mirror 6 from measurement images alone. Auxiliary metrology or a validated constrained-state model is required for a realizable extension. Given sufficient state information, Equation (1) reconstructs the imaging channel shift without requiring a structural re-solve during inference.

On the simulated dataset, the method achieved an RMSE of 0.130px for the observable correction (0.133px after INT8 quantization), compared with 0.396px for centroid-only tracking and 0.236px for the FEA-style mismatch surrogate. The measured correction error coincides with the structural scale $C_{\mathrm{sh}}\sigma_{\delta \mu }=0.132\;\mathrm{px}$ set by the common-path compensation residual, so the correction accuracy is limited by the measurement structure rather than by the capacity of the network; 22.9, 27.5, and $55.0\;\mu \mathrm{rad}\;{\mathrm{px}}^{-1}$ are the geometry-derived sensitivities that this statement rests on. Substituted into the recovery rule, the estimator gives an end-to-end imaging-shift RMSE of 0.398px, of which the displacement tracking term contributes 0.378px and the regressor only 0.130px; the end-to-end accuracy is therefore currently limited by the tracking stage, and the shared-path match must be held at the sub-percent level (Section 4.2). The full-precision network costs $9.02\times 10^{-4}$ G MACs per frame and the 80%-retained INT8 variant $6.06\times 10^{-4}$ G, i.e., 0.0150 and 0.0101ms of projected compute-only time at the assumed 120GOPS throughput—3.0% and 2.0% of the 0.5ms frame interval. These are conditional compute budget estimates. They do not demonstrate a sensor-to-output deadline, resource sufficiency, or sustained operation on flight or laboratory hardware. The error budget (24) describes the measured accuracy as the quadrature sum of the displacement tracking, auxiliary metrology, and correction-scale terms and reproduces it to within 1%. The calibration study shows that a few-percent error in the mirror-6 sensitivity costs at most 0.02px, and that auxiliary metrology below 1μrad is not a limiting term; the bit-width and pruning ablations identify INT8 with 80% of the channels retained as the smallest usable configuration of the simulated study.

The evidence is limited to synthetic opto-mechanical signals and rendered measurement frames. The study does not include an optical bench, independent ray-tracing validation, hardware-in-the-loop testing, an edge accelerator profile, or on-orbit measurements. Consequently, the reported accuracy and compute-only timing should be interpreted as feasibility results under the three stated modeling assumptions, not as demonstrated flight performance. The linear auxiliary observation baseline quantifies image non-idealities, fault gating trade-offs, covariance effects, and assumed structural model mismatch. These results support only the explicit auxiliary observation model. Future research directions include independent optical-model and laboratory validation of the augmented observations in Section 2.3, including mirror-7/8 conditioning, auxiliary sensor calibration, and comparison with the linear estimation baseline. A specified hardware implementation is needed to assess stage-level and end-to-end timing using a consistent operation count. These simulations cover simplified defocus, background gradients, gain/offset variation, exposure averaging, and specified tracking faults. Their extension to instrument-dependent thermal distortion, scattering, and detector response remains a target for physical validation. Payload-specific structural and disturbance information is also needed to select the angular operating range and calibrate the FEA comparison. These simulations quantify covariance for the linear auxiliary baseline; covariance of the original neural errors and extension beyond the small-angle regime remain additional directions. These investigations are not part of the present results and do not support claims of current physical or real-time performance.

## Figures and Tables

**Table 1:** Notation used in this paper.

Symbol	Meaning
*C*	shared elements (primary–quaternary mirrors)
*M*	measurement channel elements (switching and folding mirrors)
*I*	imaging channel elements (infrared folding mirror)
$\theta_{i}^{\prime }$	angular deviation from the calibration reference of element *i*, i∈C∪M∪I (rad)
$\Delta_{i}$	vibration sensitivity of non-shared element *i*, i∈{6,7,8} (rad px^−1^)
$S_{i}^{\left(k\right)}$	image shift sensitivity of element *i* in channel *k* (px rad^−1^)
$P_{\mathrm{img}}$	imaging channel displacement from the calibration reference (px)
$P_{\mathrm{meas}}$	measurement channel displacement from the calibration reference (px)
$f_{k}$	effective focal length of channel *k* (m)
*p*	detector pixel pitch (m)
$f_{s}$	frame rate of the measurement CMOS (Hz)

**Table 2:** Architecture and key hyperparameters of the correction regressor N. Each stage applies a 3×3 depthwise convolution (stride 2) followed by a 1×1 pointwise convolution; “Res.” is the spatial resolution after the stage.

Stage	Operation	Ch. (In→Out)	Res.	Params
Input	grayscale patch	1→1	$64^{2}$	–
1	DW3×3/s2 + PW	1→32	$32^{2}$	74
2	DW3×3/s2 + PW	32→48	$16^{2}$	1904
3	DW3×3/s2 + PW	48→64	$8^{2}$	3616
4	DW3×3/s2 + PW	64→96	$4^{2}$	6880
5	DW3×3/s2 + PW	96→128	$2^{2}$	13,376
Head	GAP + FC(128→64) FC(64→1)	128→1	$1^{2}$	8321
Total (full precision)				34,171
Total after pruning (80% retained)				23,614

**Table 3:** Estimated allocation of the 0.5 ms (2000 Hz) frame interval across the non-inference pipeline stages and the network. The times are order-of-magnitude estimates from the operand counts and the assumed rates, not measured latencies; the last row is a residual budget rather than an estimate.

Stage	Basis (Work at Rate)	Est. Time	Share
DMA transfer	$64^{2}$ px at 200 MB/s	20.5 μs	4.1%
Background removal	$∼\;2.6\times 10^{4}$ ops at 2 GOPS	13.0 μs	2.6%
Centroid localization	$1.8\times 10^{3}$ ops at 2 GOPS	0.9 μs	0.2%
Network inference (INT8)	$9.02\times 10^{5}$ MAC at 120 GOPS	15.0 μs	3.0%
Digital subtotal	—	49.4 μs	9.9%
Exposure and readout	residual frame-time allowance	≤451 μs	≤90.2%

**Table 4:** Sensitivity of shift recovery to shared-path magnification imbalance.

β [%]	Residual RMS [px]	Total RMSE [px]
0.10	0.0122	0.1306
0.25	0.0305	0.1335
0.50	0.0610	0.1436
1.00	0.1219	0.1782
1.50	0.1829	0.2244
2.00	0.2438	0.2763
3.00	0.3657	0.3884
5.00	0.6088	0.6225

**Table 5:** Accuracy on the fixed 10,000-patch test set at the nominal operating point: RMSE and MAE of the observable correction $q_{78}$, followed by RMSE of the recovered imaging channel shift $P_{\mathrm{img}}$. Brackets give 95% percentile bootstrap intervals from 10,000 resamples of samples. The oracle row uses the true measurement channel displacement and therefore isolates the structural scale of the correction.

Method	RMSE$\left(q_{78}\right)$ [px]	MAE$\left(q_{78}\right)$ [px]	RMSE$\left(P_{\mathrm{img}}\right)$ [px]
Centroid-only	0.396 [0.390, 0.403]	0.310 [0.305, 0.314]	2.622 [2.592, 2.653]
FEA-style surrogate	0.236 [0.234, 0.239]	0.196 [0.194, 0.199]	0.375 [0.370, 0.380]
Proposed (FP32)	0.130 [0.128, 0.132]	0.103 [0.102, 0.105]	0.398 [0.391, 0.404]
Proposed (INT8)	0.133 [0.132, 0.135]	0.106 [0.105, 0.108]	0.401
Oracle (true displacement)	0.131 [0.129, 0.133]	0.105 [0.103, 0.106]	0.131 [0.129, 0.133]

**Table 6:** In-envelope temporal waveform generalization tests with the full-precision network, without retraining. Conditions differ in frequency content or waveform within the trained amplitude envelope. Brackets give trajectory-level bootstrap 95% confidence intervals (20 trajectories of 400 frames).

Condition	Centroid [px]	Proposed [px]
In-envelope reference (nominal bands)	0.290 [0.284, 0.297]	0.132 [0.130, 0.134]
Unseen lower bands: 35/80/160 Hz	0.290 [0.284, 0.296]	0.130 [0.128, 0.132]
Unseen higher bands: 65/145/240 Hz	0.286 [0.281, 0.292]	0.131 [0.129, 0.132]
Mixed waveform: chirp, 280 Hz line, impulses	0.281 [0.276, 0.287]	0.130 [0.128, 0.132]

**Table 7:** Model size and projected compute-only cost. “Size” is the stored weight footprint at the stated precision; the times are operation-count/throughput estimates assuming 120 GOPS for INT8, not measured hardware latencies. The surrogate has no per-frame network cost: it requires an off-line structural solution, so its row is not applicable.

Method	Params	MACs	Size	Time	RMSE
		[G]	[MB]	[ms]	[px]
Centroid-only	0	0	0	n/a	0.396
FEA-style surrogate	n/a	n/a	n/a	off-line	0.236
Proposed (FP32)	34,171	$9.02\;\times \;10^{-4}$	0.137	0.0150	0.130
Proposed (INT8)	34,171	$9.02\;\times \;10^{-4}$	0.034	0.0150	0.133
Proposed (INT8, 80% ch.)	23,614	$6.06\;\times \;10^{-4}$	0.024	0.0101	0.147

**Table 8:** Image generation settings used for the additional studies. These settings do not replace the original network’s detector or training protocol.

Quantity	Setting
Records per condition	20 independent trajectories, 400 frames each
Sampling and exposure	2000 Hz; 0.50 ms, three midpoint sub-samples
Spot image and nominal width	24×24; Gaussian $\sigma_{0}=0.8$ px
Signal and background	5000 and 80 expected electrons per spot/frame and pixel
Read noise; clipping	3 electrons RMS; range 0–2000 electrons/pixel
Response non-uniformity	2% gain RMS; 3-electron fixed-offset RMS
Spatial background gradient	1–3 electrons/pixel per pixel along *x*
Angular input	5–13 μrad; 48–52, 115–125, 190–210 Hz
Shared-path shift	0.6 px amplitude, 37 Hz

**Table 9:** Executed image simulations for the additional studies: mean RMSE in pixels [95% interval].

Condition	Constant Background	Plane Background
Nominal	0.161 [0.157, 0.166]	0.165 [0.161, 0.169]
Uniform defocus	0.159 [0.154, 0.164]	0.163 [0.158, 0.167]
Single-lens defocus	0.162 [0.157, 0.167]	0.166 [0.162, 0.171]
Background gradient	3.319 [3.223, 3.418]	0.229 [0.225, 0.234]
Gain/offset non-uniformity	0.169 [0.163, 0.174]	0.173 [0.167, 0.179]
Combined	3.281 [3.178, 3.386]	0.237 [0.224, 0.250]

**Table 10:** Mean per-trajectory error budgets (px^2^), before gating.

Condition	Diagonal Variance	Squared Total Bias	Full/Direct MSE
Nominal	0.043601	0.000051	0.027246
Background gradient	0.043737	0.023120	0.052726
Combined	0.049668	0.025885	0.056997

## Data Availability

The simulation code and the synthetic datasets generated and analyzed during this study are available from the corresponding author on reasonable request.
